# Investigating Contrastive Pair Learning’s Frontiers in Supervised, Semisupervised, and Self-Supervised Learning

**DOI:** 10.3390/jimaging10080196

**Published:** 2024-08-13

**Authors:** Bihi Sabiri, Amal Khtira, Bouchra El Asri, Maryem Rhanoui

**Affiliations:** 1 IMS Team, ADMIR Laboratory, Rabat IT Center, ENSIAS, Mohammed V University in Rabat, Rabat 10000, Morocco; bouchra.elasri@um5.ac.ma; 2 LASTIMI Laboratory, EST Salé, Mohammed V University in Rabat, Salé 11060, Morocco; amal_khtira@um5.ac.ma; 3 Laboratory Health Systemic Process (P2S), UR4129, University Claude Bernard Lyon 1, University of Lyon, 69100 Lyon, France; mrhanoui@esi.ac.ma; 4 Meridian Team, LYRICA Laboratory, School of Information Sciences, Rabat 10100, Morocco

**Keywords:** contrastive learning, supervised learning, unsupervised learning, representation learning, contrastive loss, similarity metric, unsupervised pretraining

## Abstract

In recent years, contrastive learning has been a highly favored method for self-supervised representation learning, which significantly improves the unsupervised training of deep image models. Self-supervised learning is a subset of unsupervised learning in which the learning process is supervised by creating pseudolabels from the data themselves. Using supervised final adjustments after unsupervised pretraining is one way to take the most valuable information from a vast collection of unlabeled data and teach from a small number of labeled instances. This study aims firstly to compare contrastive learning with other traditional learning models; secondly to demonstrate by experimental studies the superiority of contrastive learning during classification; thirdly to fine-tune performance using pretrained models and appropriate hyperparameter selection; and finally to address the challenge of using contrastive learning techniques to produce data representations with semantic meaning that are independent of irrelevant factors like position, lighting, and background. Relying on contrastive techniques, the model efficiently captures meaningful representations by discerning similarities and differences between modified copies of the same image. The proposed strategy, involving unsupervised pretraining followed by supervised fine-tuning, improves the robustness, accuracy, and knowledge extraction of deep image models. The results show that even with a modest 5% of data labeled, the semisupervised model achieves an accuracy of 57.72%. However, the use of supervised learning with a contrastive approach and careful hyperparameter tuning increases accuracy to 85.43%. Further adjustment of the hyperparameters resulted in an excellent accuracy of 88.70%.

## 1. Introduction

Contrastive learning is one of the most interesting and promising new discoveries in the field of artificial intelligence (AI). It has evolved as a potent machine learning technique that uses the power of comparisons to improve representations and enable unsupervised learning. This technique is a powerful machine learning approach that aims to extract meaningful representations by using similarities and differences across datasets. It has received significant attention and achieved great success in a variety of disciplines, including computer vision, natural language processing (NLP) or sentence representation learning, and recommendation systems [1].

Contrastive learning attempts to learn a robust and discriminative feature space by contrasting positive and negative pairs of samples. In this article, we investigate a comparative study based on experimentation between the contrastive learning model and conventional classification models and contrast learning bases, looking at its origins, difficulties, and significant contributions to the field of artificial intelligence. First, we examine the theoretical underpinnings of contrastive learning and consider its potential applicability to various problem domains. The basic ideas of contrastive learning will be covered first, followed by a discussion of its uses in numerous fields.

### 1.1. Understanding Contrastive Learning

#### Theoretical Foundations

- Similarity Measurement:
  The idea of calculating the degree of similarity between data samples is the foundation of contrastive learning. A common method for achieving this is to embed data points into a high-dimensional space [2,3], which pushes different samples apart and brings like samples closer together.
- Contrastive Loss Functions:
  The contrastive loss function, which penalizes the gap between similar samples and encourages a margin between different ones, is the fundamental component of contrastive learning. Decibel Contrastive Estimation (InfoNCE) [4,5,6] and Triplet Loss [2,7] are two often used contrastive loss functions. Their foundation is the idea that mutual information should be maximized for positive couples and minimized for negative ones. As the model is trained, it learns how to reduce the InfoNCE loss by adjusting its parameters. Like samples are grouped together and dissimilar samples are grouped together in the representation space, making the model’s representations more discriminative as a consequence. Through contrastive learning, similar samples are mapped to adjacent locations in the representation space by optimizing the agreement between positive pairs. Because of this feature, important patterns and similarities in the data can be captured by the learned representations even in the absence of explicit labeling. As a result, a variety of downstream tasks like categorization, detection, and retrieval can be performed using these representations. Other functions are explained in Section 4.
- Siamese Networks and Beyond:
  Originally developed for signature verification, Siamese networks are now extensively used in contrastive learning [8]. These networks are made up of two identical subnetworks that share weights so that they can acquire similarity-capturing embeddings. With the use of self-supervised methods like momentum contrast and SimCLR (Simple Contrastive Learning), contrastive learning has advanced beyond Siamese designs in recent times.

### 1.2. Applications of Contrastive Learning

- Image Representation Learning:
  When learning semantically meaningful representations from unlabeled visual data, contrastive learning has demonstrated impressive performance. Contrastive learning can acquire rich representations required for subsequent tasks such as object detection and picture classification [9,10,11] by optimizing similarity between various augmentations of the same image and decreasing it for different images.
- Text Embeddings:
  Text embeddings have been learned by contrastive learning in the field of natural language processing [12]. Contrastive learning can capture semantic interactions between text segments by taking into account positive pairs (e.g., distinct textual illustrations for the same phrase) and negative pairs (e.g., contrasting sentences). This makes tasks like question answering, sentiment analysis, and document similarity easier to complete.
- Object Recognition and Segmentation:
  It is possible to acquire visual characteristics that are helpful for object recognition through contrastive learning [13]. The model learns to encode local and global visual information by contrasting different regions within the same image. This can enhance the efficiency of subsequent tasks that need precise location and classification, such as object detection.
- Instance Discrimination:
  In instance discrimination, when the objective is to discern between various instances of the same object, contrastive learning can also be used [14]. The model learns to interpret fine-grained details and object-specific properties by comparing positive pairs of instances from various perspectives or augmentations. For tasks like object tracking or instance segmentation, this can be helpful.
- Audio and Speech Processing:
  Learning representations from voice and audio data has also been accomplished through the use of contrastive learning techniques [15]. Through the use of positive and negative pairs, such as distinct audio clips from the same speech segment, contrastive learning is able to extract relevant characteristics that are helpful for sound classification, speaker identification, and speech recognition.
- Healthcare and Biology:
  In fields such as biology and healthcare, where access to labeled data is frequently costly and datasets frequently have sparse and unbalanced data [16], contrastive learning is a viable path for representation learning [12,17]. Contrastive learning can be used to identify meaningful patterns and associations in unlabeled medical images, genomic sequences, or protein structures. This can help with tasks like disease diagnosis, medication development, and customized medicine.
- Generative Modeling:
  Contrastive learning has also been utilized in generative modeling projects, which aim to learn a probability distribution from input data [18]. Contrastive learning, by learning representations that capture relevant properties of data distribution, can assist enhance the quality of samples produced in generative adversarial networks (GANs) and other generative models.
- Domain Adaptation and Transfer Learning:
  Contrastive learning has been used in domain adaptation and transfer learning tasks [19], where the goal is to transfer knowledge from a source domain with a large amount of labeled data to a target domain with less labeled data. Contrastive learning can increase generalization performance on the target domain by teaching representations that capture domain-invariant properties.

Through comprehension of the theoretical underpinnings and investigation of its varied applications in various problem domains, we may effectively leverage contrastive learning to tackle pragmatic issues and stimulate innovation in the field of machine learning and beyond.

**Figure 1:**
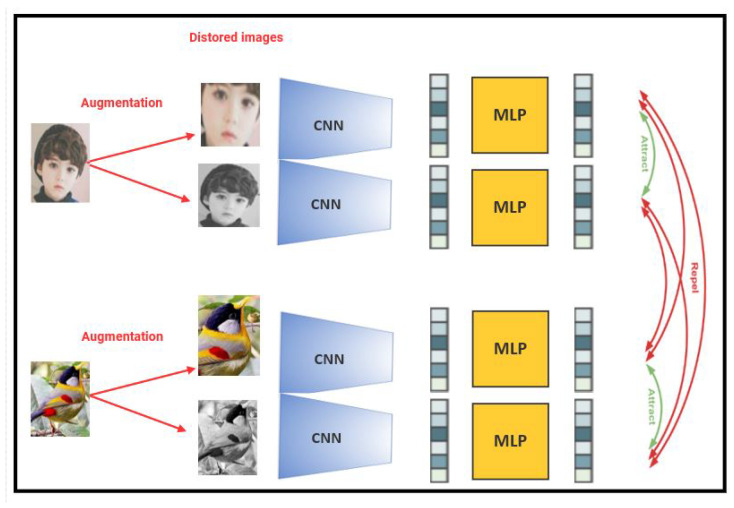
Classification of contrastive self-supervised images.

This learning approach emulates how humans learn about the world and has gained growing importance in computer vision research due to its promising results in the deep learning literature. In contrastive learning, unlabeled data points are compared with one another to train a model to distinguish which points have similarities and which do not [2] (see Figure 1).

By using the strategy of contrastive pair learning, the objective of the model is structured to find similarities and differences, much like classification tasks do when given labels. Contrastive approaches, as their name implies, instruct representations by contrasting positive and negative examples. Using this technique, the model learns representations that effectively capture the underlying semantics and discriminative properties of the data by presenting it with properly designed pairs.

Contrastive learning relies on three different learning contexts: supervised, self-supervised, and semisupervised. Self-supervised learning is a machine learning technique in which a model learns representations or features from unlabeled data without requiring explicit human supervision [7,8]. Traditional supervised learning involves training a model on labeled data, with each input paired with a corresponding target or label. However, in self-supervised learning, the goal is to derive usable representations or features from the input data. The main idea behind self-supervised learning is to create a pretext task that can be autonomously constructed from unlabeled data. This pretext challenge creates a supervised learning problem in an automated method. The model is trained to solve the pretext challenge and extract meaningful representations/features, and in the process, it learns to extract relevant features that can be used for subsequent jobs. One typical method in self-supervised learning is to employ data augmentation techniques to create pairs of enriched versions of the same input [11,20,21]. The model is then taught to predict the relationship between the upgraded versions. For instance, in image-based self-supervised learning, the model can be trained to anticipate the enhanced images’ relative position, rotation, or colorization. Self-supervised learning has received a lot of interest in recent years, particularly in the fields of computer vision [22] and NLP [23]. It has been demonstrated to be useful in pretraining models on vast volumes of unlabeled data, which may then be refined on smaller datasets with labels for particular tasks.

Supervised contrastive learning is a form of machine learning that uses information about similarity and dissimilarity across training instances to learn high-quality data representations [7]. In common supervised learning, the goal is to train a model that can correctly predict labeling classes for new unlabeled samples [23]. However, in supervised contrastive learning, the emphasis is on learning data interpretations rather than explicitly predicting class labels. The primary goal of supervised contrastive learning is to increase the similarity among representations of positive examples (from the same class) while limiting the similarity between representations of negative examples (from separate classes) [22]. This is often accomplished by presenting pairs of examples, each consisting of a good and a negative example. Supervised contrastive learning has been widely utilized in NLP and computer vision to develop high-quality representations that can then be applied to specific tasks such as image classification or phrase translation.

Semisupervised contrastive learning approaches, or SSL, are becoming more and more popular in the machine learning area [24,25,26,27,28]. SSL is an expansion of supervised contrastive learning that uses both labeled and unlabeled data to develop representations [9]. In conventional supervised learning, the model is trained simply on labeled samples. Semisupervised learning utilizes mixed tagged and unmarked examples [29]. The primary reason for semisupervised contrastive learning is to make use of the amount of unlabeled data, which is typically more easily obtained than labeled data [30]. Including unlabeled instances during training allows the model to acquire more robust and generalizable representations [20]. The method often entails creating contrastive pairs or collections of labeled and unlabeled samples. The goal is to increase the similarity between representations of positive examples within the same class while decreasing the similarity between representations of negative examples from different classes (see Figure 1). This promotes the model to acquire representations that include both class-specific information from labeled instances as well as significant structure from unlabeled examples.

Semisupervised contrastive learning has produced promising results in a variety of fields, including healthcare, computer vision, and natural language processing [18,31].

Our primary findings and contributions can be summarized as follows:

- Experimental methods verify that the contrast-based model, by employing contrast approaches, may effectively capture significant representations by identifying the similarities and differences between modified versions of the same image. This capability surpasses that of traditional categorization approaches. Furthermore, this experimental evidence supports this assertion for both the self-supervised and semisupervised training models as well as the supervised training model.
- In our semisupervised learning research, we identified practical and effective strategies for identifying probable false negatives through contrastive learning. These methods are simple to apply and have shown a notable accuracy of around 57.72% in detecting false negatives, even when only 5% of the data are labeled. This shows that our approach could be useful for detecting misclassified instances and boosting the model’s overall performance in circumstances with minimal labeled data.
- We demonstrate how the temperature is a crucial factor in determining the severity of penalties on hard negative samples using a supervised contrastive loss weighted by the temperature and base temperature.
- For contrastive learning with an encoder, large batch sizes and large-scale datasets are essential; otherwise, optimal results are difficult to achieve.
- When supervised learning is paired with a contrastive approach and thorough hyperparameter adjustment, significant accuracy gains are obtained. This synergistic technique improves model performance by combining the benefits of supervised and contrastive learning. The careful adjustment of hyperparameters helps to achieve optimal outcomes. Overall, this combined methodology demonstrates an excellent strategy for improving accuracy in the current context.
- The accuracy and meaningful representation capture of deep image models are greatly enhanced by contrastive techniques in conjunction with careful hyperparameter adjustment.

The following is how this paper is structured: Section 2 goes into further detail about the pertinent studies and research in the field. In Section 3, we discuss Attractive–Repulsive Dynamics and Applications of Contrastive Learning. Following that, we present underpinning contrastive learning mathematically in Section 4. Section 5 explains our approach, while Section 6 describes in detail the experimental set-up and the results obtained. In Section 7, we discuss and evaluate various limitations of the method. The Section 8 contains some conclusions and proposals for future investigation.

## 2. Related Works

Without claiming to be exhaustive, we list a few correlations between contrastive leaning and earlier research below:

### 2.1. Image Classification

The goal of the study by [20] is to investigate the application of universum-style Mixup in the context of supervised contrastive learning to produce high-quality hard negatives, which can increase the resilience and adaptability of neural network training. The article introduces Universum-inspired Contrastive Learning (UniCon), which uses the Mixup technique to produce universum data as hard negatives and separate them from anchor samples of the target classes. The goal is to improve Mixup with solid labels and develop a new metric for generating universal data.

### 2.2. Batch Contrastive Approaches

In [7], the authors suggest a modification of the self-supervised batch contrastive technique that may be used in the fully supervised environment, allowing for the efficient use of label information. Their results consistently show that this method beats cross-entropy on a variety of datasets and ResNet variations, and it provides benefits in terms of robustness to spontaneous corruptions and stability to hyperparameter settings.

### 2.3. Enhanced Image Rotation

A model is trained in the challenge to categorize the rotation degree of input images (such as 0°, 90°, 180°, and 270°) [32,33]. Because rotation entails identifying the correct orientation of objects in input photos, models that successfully complete the task can learn knowledge about object shapes (such as the silhouette of cats). Using a pretraining task like the rotation task, SSL is a promising method for learning image representations for vision tasks. It then uses these representations to perform the target vision tasks.

### 2.4. Enhanced Text Classification by Self-Learning

Training a deep learning text categorization model often requires a large amount of labeled data, which is expensive and time-consuming [12]. However, few-shot text classification attempts to predict unknown samples using a small number of identified data. There has been a recent surge of interest in metric-based approaches to few-shot text classification. This was made better by metric-based metalearning, which increased generalization during episodic training. Existing models, however, ignore learning from a large number of unlabeled samples. This research exploits model information from unlabeled samples to improve the generalization of a metanetwork. It introduces knowledge distillation, enhancing metalearning with self-supervised data, and a way of aggregating graphs for representations that are more discriminating. Improved model performance has been shown in experiments using datasets for few-shot text classification.

### 2.5. Improved Detection of Electricity Theft Using Supervised Contrastive Learning

Using supervised contrastive learning [23,34], a novel approach to electricity theft detection (ETD) is put forth. The technique actively compares the representation vectors of users to enhance detection performance. For high-quality augmented views, the largest triangular three buckets time series downsampling is used.

### 2.6. Getting Rid of Redundancy in Self-Supervised Learning

Barlow Twins proposes an objective function for self-supervised learning that eliminates redundancy across embedding vector components by assessing the cross-correlation matrix between the outputs of two related networks fed with distorted copies of a sample [8].

### 2.7. Optimized Clustering in Semisupervised Leaning

In the semisupervised learning of neural networks, the cost function encourages compact clustering of the latent space for better separation. Over embeddings of labeled and unlabeled samples, a graph is constructed, and a Markov-chain-based cost function controls the latent space to build a single compact cluster for each class. This method combines effective, inductive inference with graph-based regularization without changing the network architecture. The suggested approach is tested against three benchmarks and shows encouraging results, making it a possible remedy for the efficient use of unlabeled data in current networks [35].

### 2.8. Weakly Supervised Contrastive Learning

The authors in [36] proposed Simple Weakly Supervised Contrastive Learning (SWCL) for sentiment classification in an unsupervised setting. They use label information to combine supervised contrastive learning and fine-tuning. They presented a contrastive loss function extension and used BERT as the SWCL encoder. The framework consists of unsupervised contrastive pretraining followed by supervised fine-tuning on a small labeled dataset.

SWCL achieves cutting-edge performance on downstream NLP tasks by using powerful representations learned by pretrained BERT models. The authors also talked about transfer-style weakly supervised contrastive learning and a multitask joint learning technique that produces results comparable to a two-step transfer learning approach.

SWCL’s overall design entails BERT-encoding weakly labeled text and contrastive learning in the embedding layer to capture sentiment patterns.

## 3. Requirements and Principles of Contrastive Learning

### 3.1. Contrastive Learning

With its ability to capture both similarities and differences between data samples, contrastive learning can be appealing not just in attracting comparable instances but also in repelling different cases (See Figure 1). This attractive–repulsive quality of contrastive learning is an important component that contributes to its success in learning meaningful representations. The capacity of contrastive learning to bring similar occurrences closer together in the learned representation space is referred to as its “attractive” feature. Contrastive learning facilitates the embedding of these examples in close proximity to one other by training the model to reduce the distance between positive pairings that consist of similar occurrences. This proximity allows for improved discrimination and allows the model to capture shared traits and patterns among comparable occurrences. Contrastive learning, on the other hand, possesses a “repulsive” quality that separates different instances in the learned representation space. Contrastive learning allows the model to learn representations that push apart examples that do not share common properties by maximizing the distance between negative pairings made up of different instances. This repulsion ensures that dissimilar instances are adequately separated in the embedding space, making downstream tasks easier to distinguish between distinct classes or categories.

The attractive–repulsive aspect of contrastive learning is critical to its success in a variety of applications. In computer vision, for example, contrastive learning can attract multiple views of the same object while repelling views of distinct objects. This enables the model to build robust representations that capture intraclass similarities and interclass differences, resulting in improved object recognition and classification performance.

**Figure 2:**
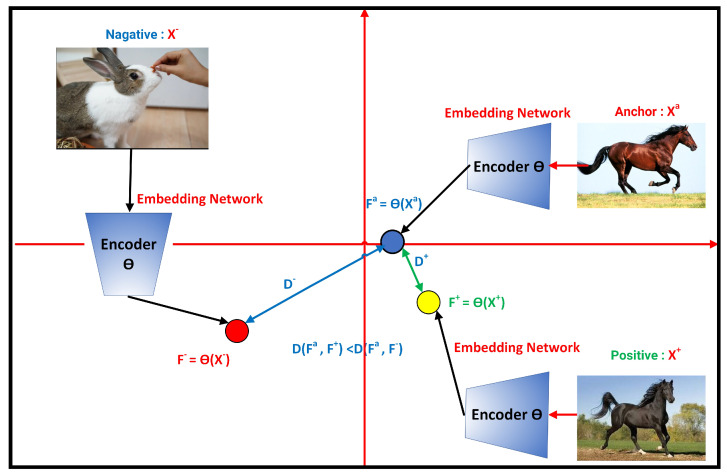
The contrastive learning model’s purpose is to increase the distance $D^{-}$ while minimizing the distance $D^{+}$.

Formally, contrastive techniques look for an encoder Θ that meets the following requirements:

1. A positive sample, represented by the symbol $X^{+}$,
  is a data point that is identical to $X^{a}$ ($X^{a}$ is a symbol of the anchor (an anchor refers to a reference or target and a baseline to measure the similarity of other data points within a dataset) ($X^{+}$ is similar to $X^{a}$). It can be an image of the same class as the anchor or an augmented version of the anchor’s image;
2. A negative sample, represented by the symbol $X^{-}$, is a data point that is different from $X^{a}$ ($X^{-}$ is dissimilar to $X^{a}$);
3. The score function F is a metric that evaluates how closely two features resemble each other, and the inequality specified below has to be satisfied (See Equation (3) and Figure 2).

Regardless of the initial distances between the anchor sample and the positive sample D$(\left(X^{a}\right),\left(X^{+}\right)$) and between baseline sample and negative sample D$(X^{a},X^{-}$), the purpose of contrastive learning is to encourage the model to have a smaller distance between an anchor sample and a positive sample than between the anchor sample and a negative sample (See Equations (1) and (2) and Figure 2). Similar occurrences are embedded closer together in the learned representation space, whereas dissimilar instances are pushed further apart.

(1) $$D\left(\theta \left(X^{a}\right),\theta \left(X^{+}\right)\right)<D\left(\theta \left(X^{a}\right),\theta \left(X^{-}\right)\right)$$

By replacing $\theta (X^{a})$ by $F^{a}$, $\theta (X^{+})$ by $F^{+}$, and $\theta (X^{-})$ by $F^{-}$, the previous equation becomes

(2) $$D\left(F^{a},F^{+}\right)<D\left(F^{a},F^{-}\right)$$

or simply by replacing $D(F^{a},F^{+})$ with $D^{+}$ and $D(F^{a},F^{-})$ with $D^{-}$ (See Figure 2).

(3) $$D^{+}<D^{-}\;$$

### 3.2. Components That Contribute to Contrastive Learning

#### 3.2.1. Predicting an Image’s Rotation

**Figure 3:**
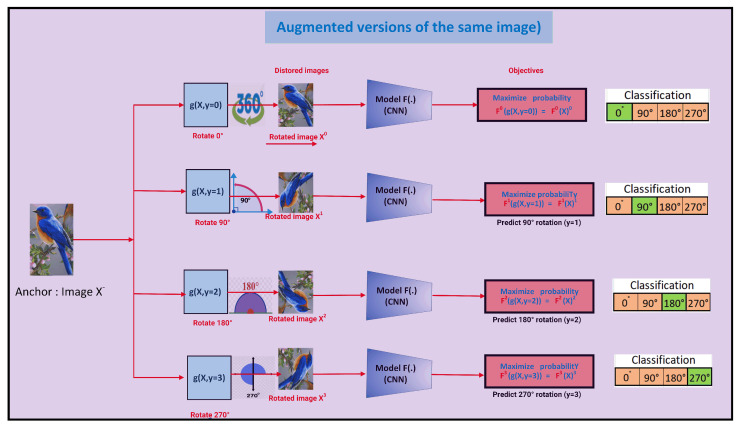
The learning of self-supervised representations from rotating input images.

Every input image is initially randomly rotated by a multiple of 90° [33,37]. To forecast the amount of rotation used, a model is trained [8]. Although it is a four-class classification issue in its simplest form, several variations can be thought of. This pretext task forces the model to learn the semantic components of objects, such as arms, legs, and eyeballs, in order to estimate the amount of rotation. As a result, it might be useful for a task that comes after, like object recognition (see Figure 3). Rotations are examples of Euclidean isometries (transformations that keep distance intact) that preserve an object’s original shape [37]). This property of isometry (shape-preserving modifications) improves the learning of shapes by allowing the following tasks, such as classification, to focus on collecting a single label for each shape rather than requiring the acquisition of labels for each unique training instance. As shown in Figure 3, for reliable prediction, models are trained to be rotation-invariant because rotating an image does not change the essential information it contains (a bird in the image will still be a bird, for example).

#### 3.2.2. Discrimination Method

**Figure 4:**
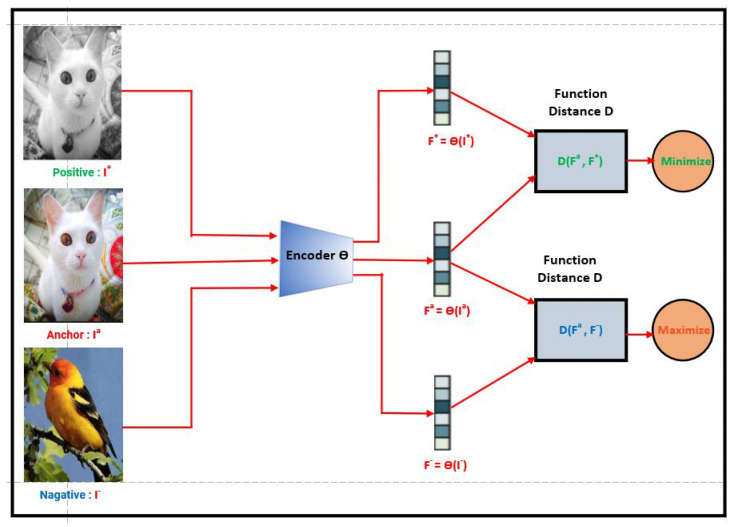
Discrimination method.

In this type of contrastive learning, each and every image is altered before being utilized as a positive example for an anchor image. If we choose to use an image of a cat as the anchor, for instance, we may either color it, mirror it, or convert it to grayscale to use as the positive sample. The negative sample can be any additional image from the collection (See Figure 4).

#### 3.2.3. Contrastive Losses between Self-Supervised and Supervised Learning

**Figure 5:**
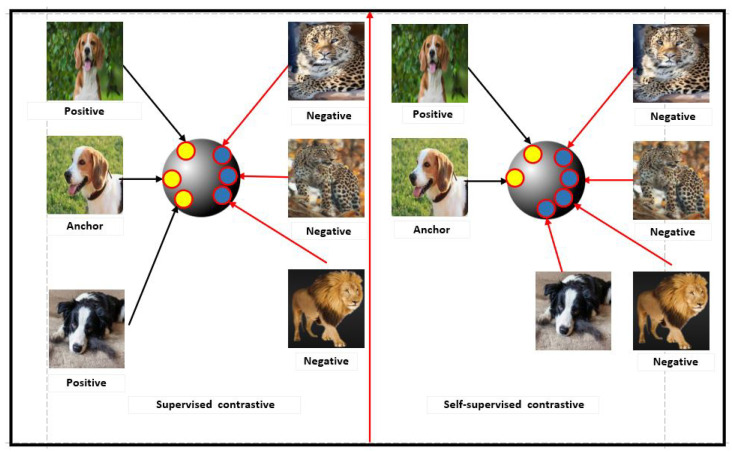
Losses comparing supervised and unsupervised techniques. Supervised contrastive learning considers many samples from the same class as positive examples in addition to augmented versions. The colors of the arrows distinguish between the two types of images: positive and negative.

The supervised contrastive loss method compares all samples within the same class as positive examples against the negatives from the remaining set. In contrast, the self-supervised contrastive loss approach compares a single positive example for each anchor (which is an augmented version of the same image) against a set of negatives consisting of all the other instances in the mini-batch (See Figure 5).

## 4. Mathematical Basis of Error Calculation in Contrastive Learning

**Figure 6:**
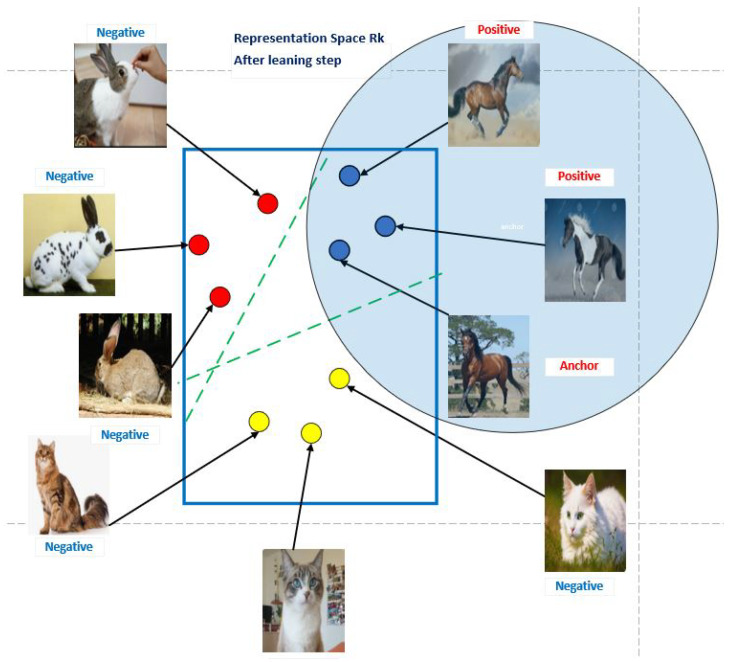
Adjusting image embeddings proximity. The colored circles define a type of categorization in the embedding space, projecting the objects into distinct zones based on their classification.

The neural network’s output in deep metric learning is an embedding representation of the input rather than a one-hot encoded vector or a softmax output, as it is in conventional classification networks. The network learns how to use these embedding representations to construct clusters of instances from each class by spacing inputs from the same class apart in embedding space and maintaining a buffer between the various classes. The development of an appropriate loss function is a crucial step in metric learning since it promotes the global minimum search and allows for quick convergence. According to the graphic in Figure 6, the concept behind contrastive learning is that we have three samples: an anchor sample, a sample that is comparable to or “positive”, and a sample that is different from or “negative”. In an embedding space, we attempt to push negative samples apart while bringing positive samples closer to the anchor sample.

In the computer vision example, we might try to develop an encoder that pushes positive image embeddings together and negative embeddings away (for example, a convolutional neural network). An image from the same class as the anchor or an enhanced version of the anchor image may be a positive sample, but an entirely different image (often from a different class) would be a negative sample. In this illustration (Figure 6), we move the embedding from the cat and rabbit image further away while pushing the embedding from the identical horse image closer together (See Figure 6).

There are numerous suggestions for loss functions, all of which are based on the Euclidean distance between the training inputs in embedding space, such as contrastive loss, triplet loss, and multiclass N-pair loss.

Different loss functions have been defined to train models efficiently in the setting of contrastive learning. In the literature on contrastive learning, the following loss functions are frequently employed.

### 4.1. InfoNCE

InfoMax Contrastive Estimation (NCE is an acronym for noise-contrastive estimation) (refs. [8,38,39,40]) is based on the idea that mutual information should be maximized for positive pairings of samples while being minimized for negative pairs. It frequently functions in tandem with the contrastive predictive coding system. We take into account a set X = ($x_{1},\ldots x_{N}$) made up of N random samples from $p(x_{\left(t+k\right)}|c_{t})$ in order to maximize $L_{N}$. This set consists of N − 1 negative samples taken from the ’proposed’ distribution $p(x_{(t+k})$. Our objective is to increase $L_{N}$’s value through the process of learning:

(4) $$L_{N}=-E_{X}\left[\log\frac{f_{k}\left(x_{t+k},c_{t}\right)}{\sum_{x_{j}\in X}f_{k}\left(x_{j},c_{t}\right)}\right]$$

If this loss is optimized, the density ratio will be estimated by $f_{k}\left(x_{t+k},c_{t}\right)$ as

(5) $$f_{k}\left(x_{t+k},c_{t}\right)\propto \frac{p\left(x_{t+k}|c_{t}\right)}{p\left(x_{t+k}\right)}$$

The generative model $p(x_{t+k})$ preserves the mutual information between the future observations $x_{t+k}$ and the context latent representation $c_{t}$ through the density ratio, where the following hold:

- $L_{N}$: This is a measure of the difference between predicted and true probabilities in the context of contrastive learning (loss).
- $E_{X}$: This is the expected loss over the dataset X, calculated by taking the average of all cases.
- $x_{t+k}$: This is the augmented view (future observation) of an instance at time t + k.
- $f_{k}$: This represents the similarity function between augmented views. It compares the resemblance or agreement between $x_{t+k}$ and $c_{t}$.

Equation (4) can be expressed in terms of $x^{+}$ (positive) and $x^{-}$ (negative), as follows:

(6) $$L_{\mathrm{InfoNCE}}=-\log\frac{\exp\left(\mathrm{sim}\left(x^{a},x^{+}\right)/\tau \right)}{\sum_{x^{-}}\exp\left(\mathrm{sim}\left(x^{a},x^{-}\right)/\tau \right)}$$

The anchor, positive, and negative embeddings in this equation are denoted by $x^{a}$, $x^{+}$, and $x^{-}$.

- $sim\left(x^{a},x^{+}\right)$: This is the similarity score between the anchor and positive samples. The implementation determines the specific similarity function used (e.g., dot product or cosine).
- $sim\left(x^{a},x^{-}\right)$: This is the similarity score between the anchor and negative samples.
- τ: The temperature parameter determines the smoothness of the probability distribution (see Section 5.2.1).

As is typical in the self-supervised environment, we have one positive sample and several negatives (N − 1).

We broaden the equation in the supervised situation to take into account multiple positive samples. The vector cosine similarity function is known as the sim function.

According to [41], we want to maximize the cosine similarity between $x^{a}$ and $x^{+}$, which will bring them closer together. For $x^{a}$ and $x^{-}$, the opposite is accurate. A temperature hyperparameter is also available, and it regulates the penalty for tougher negative samples. Harder negatives are penalized more severely at lower temperatures.

### 4.2. Normalized Temperature-Scaled cross Entropy Loss

NT-Xent (Normalized Temperature-scaled Cross Entropy) [12,42]: The NT-Xent loss is an adaptation of the cross-entropy loss that includes temperature scaling and normalization. It encourages the model to provide more points for similarity to positive pairs and fewer points for similarity to negative pairs. To put it simply, 2N images that come from N underlying images are delivered to the contrastive learning model. Two augmented images are created from each of the N underlying photos using a random collection of image augmentations. On account of this, we are able to feed the model 2N photos in a single train batch. The loss function for a pair of positive samples $\left(z_{i},z_{j}\right)$ is then

(7) $$L_{z_{i},z_{j}}=-\log\frac{\exp\left(\mathrm{sim}\left(z_{i},z_{j}\right)/\tau \right)}{\sum_{k=1}^{2N}1_{\left[k\neq i\right]}\exp\left(\mathrm{sim}\left(z_{i},z_{k}\right)/\tau \right)}$$

where $\mathrm{sim}\left(a,b\right)=a^{T}b/\left||a||||b|\right|$ indicates cosine similarity of two vectors a and b, τ is a temperature scalar (See Section 5.2.1), and $1_{\left[k\neq i\right]}\in \left[0,1\right]$ is a signaling function that evaluates to 1 if k≠i.

### 4.3. Additional Equations for Contrastive Error Learning

Other losses are employed in the contrastive learning model training process. The Table 1 summarizes them.

**Table 1:** Diverse loss functions in contrastive learning.

Loss	Equation
Logistic Loss [43,44,45]	(8) $J\left(\theta \right)=\frac{-1}{N}\sum_{i=1}^{N}y^{\left(i\right)}\log\left(\theta \left(x^{\left(i\right)}\right)\right)+\left(1-y^{\left(i\right)}\right)\log\left(1-\theta \left(x^{\left(i\right)}\right)\right)$
Max margin Contrastive Loss [46]	(9) $L\max-\mathrm{margin}=\left(\sum_{j=1}^{N}1_{\left[y_{i}=y_{j}\right]}\cdot d\left(x^{i},x^{j}\right)-\sum_{j=1}^{N}1\left[y_{i}\neq y_{j}\right]\cdot \max\left(0,m-d\left(x^{i},x^{j}\right)\right)\right)$
Triplet Loss [47]	(10) $L_{\mathrm{triplet}}=\sum_{i=1}^{N}\left[\max(0,d\left(x^{a},x^{+}\right)-d\left(x^{a},x^{-}\right)+\mathrm{margin})\right]\;$
N-pair Loss [48,49] (See Figure 7)	(11) $L_{N-\mathrm{pair}}=\sum_{i=1}^{N}\log\left(1+\sum_{j=1}^{N-1}\exp{\left(\theta (x^{a}\right)}^{T}\cdot \theta \left(x_{i}^{-}\right)-\theta {\left(x^{a}\right)}^{T}\cdot \theta \left(x^{+}\right)\right)\;$

## 5. Materials and Methods

We first evaluated traditional data categorization methods and compared them to the contrastive learning framework. This enabled us to test the performance of standard frameworks and compare their results to contrastive learning. After evaluating the contrastive learning framework’s superior performance, we moved on to the next stage, which included data augmentation, training an encoder network, and fine-tuning the various hyperparameters to improve performance. Following that, we moved on to the evaluation and generalization processes, which are critical for determining the quality of acquired representations and their efficacy in real-world applications. To improve the results, hyperparameters such as batch size, learning rate, latent space dimension, and loss function were changed and tuned. By adjusting these hyperparameters, we hoped to improve the framework’s ability to capture important differences across samples and deliver more accurate and discriminative results. This customization method entailed iterative experimentation and evaluation of the framework’s performance using relevant evaluation criteria like accuracy or F1 score. The purpose of this tuning step was to determine the ideal hyperparameter configuration that would result in considerable performance increases in contrastive learning. By fine-tuning the system, we hoped to capitalize on its natural strengths, such as catching intrinsic data patterns and generalizing well in cases with minimal labeled data. Subsequently, by adjusting the hyperparameters, we hoped to maximize the contrastive learning framework’s potential and improve its performance in data categorization tasks.

Afterward, we delved into the realm of contrastive learning, which we recommend using in self-supervised, supervised, and semisupervised learning scenarios.

In self-supervised learning [50,51], where labeled data are rare or unavailable, contrastive learning can be used to develop meaningful representations from unlabeled data. Contrastive learning allows the model to capture underlying patterns and structure in the data by training it to increase the agreement between multiple enhanced views of the same instance and limit the agreement between views of different instances. With little labeled data, this learned representation can subsequently be used for downstream tasks like classification or clustering.

In semisupervised learning [10,35], where a small amount of labeled data are accessible with a large amount of unlabeled data, contrastive learning can be utilized to improve learning. By including contrastive learning loss in addition to typical supervised loss, the model can benefit from both labeled and unlabeled data. The contrastive loss enables the model to capture similarities and differences across examples, which improves the generalization and discriminating skills of the learned representations. This can lead to improved performance, especially when the labeled data is restricted.

Contrastive learning can still be useful in supervised learning [7] when there is a lot of labeled data available. The model can learn more robust and informative representations by including contrastive learning as an auxiliary task. This can increase the model’s generalization capabilities and performance on the supervised learning task.

### 5.1. Pairwise Ranking Loss

In contrastive learning, pairwise ranking loss refers to a loss function that is used to train a model to compare and rank pairs of samples [52]. The purpose of contrastive learning is to acquire representations that can differentiate between positive pairs (similar samples) and negative pairs (dissimilar samples). The pairwise ranking loss is intended to encourage the model to assign higher ranks to positive pairs and lower rankings to negative pairs.

This approach is analogous to the preference learning utilized in RankNet [53], where a binary classifier is used to compare pairs of samples and produce a rating based on their relative preferences. The classifier’s output nodes use softmax activation, and a threshold is employed to assess whether the absolute difference between the mean arousal levels of two segments is large enough to consider them as a preference pair for training. This method generates a balanced dataset for deep preference learning.

**Figure 7:**
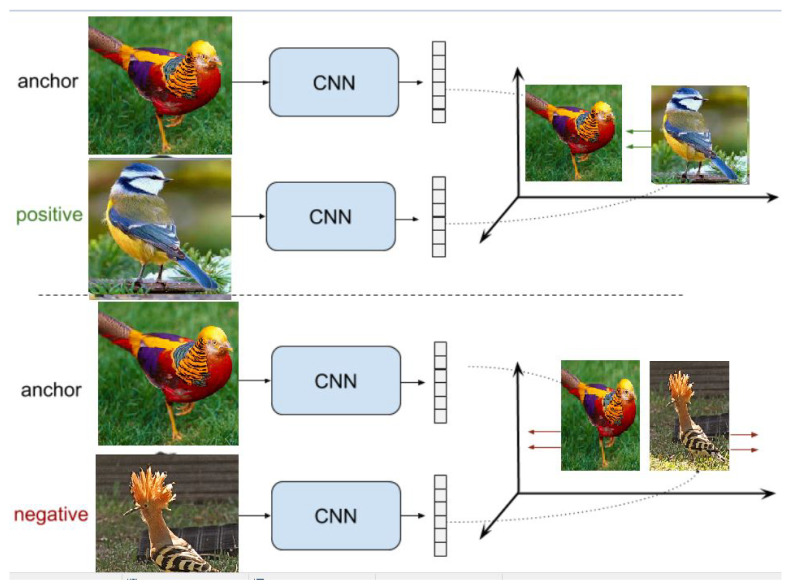
Pairwise ranking: The arrows on the left indicate the transformation from image to vector representation. The arrows on the right represent the convergence of similar images and the divergence of dissimilar images in the vector space.

The pairwise ranking loss is a typical loss function used in contrastive learning to train models for tasks such as image similarity or recommendation systems. It assists the model in learning to differentiate between similar and dissimilar data by penalizing erroneous rankings and rewarding correct rankings (See Figure 7).

### 5.2. Hyperparameters

#### 5.2.1. Temperature

Temperature refers to the degree of originality and randomness in the generated result [54]. It is a crucial hyperparameter that describes the “softness” of the softmax distribution utilized in the cross-entropy loss. Higher contrastive accuracy often results from lower values. The value of the temperature can also be learned using a recent align technique, which involves defining the temperature as a variable and using gradients to change its value. This gives a reasonable baseline value, but because it is tuned for contrastive loss, which is not a perfect proxy for representation quality, the learned temperature in our trials was a little below ideal. The output of a neural network is frequently transformed into a probability distribution over several classes using the softmax function. It determines the output logits of the network’s exponentiated values and normalizes them to sum to 1. The temperature parameter, commonly abbreviated as “τ”, is added to alter the probability distribution’s sharpness or smoothness. The output logits of the softmax function are exponentiated after being split by the temperature parameter when temperature scaling is used. The probability distribution that results from raising the temperature is smoother and more evenly distributed among the classes. On the other hand, lowering the temperature produces a sharper distribution where the probabilities are concentrated in a small number of classes.

When performing activities that need self-supervised learning, such as contrastive learning or generative modeling, temperature scaling might be helpful. Through improved latent space exploration during training, more varied representations can be learned by the model. In accordance with the precise goals of the self-supervised learning task, the temperature parameter can be changed to manage the degree of discrimination or diffusion in the learned representations.

#### 5.2.2. Strength of the Image Augmentation

During pretraining, stronger augmentations make the work harder, but after a certain point, too strong augmentations will hurt performance. Stronger augmentations avoid overfitting during fine-tuning, but in the authors’ experience, too strong augmentations lessen the performance benefits of pretraining.

#### 5.2.3. Optimizer

Adam is the optimizer chosen in this example since it performs well with the default settings. Although SGD with momentum takes more fine-tuning, performance might be marginally improved.

#### 5.2.4. Learning Rate Schedule

Although a cosine decay plan, which might further enhance performance, is frequently employed in the literature, a constant schedule is chosen here.

### 5.3. Algorithms

A machine learning technique called self-training for semisupervised image classification uses labeled and unlabeled data to enhance the performance of image classification models. When labeled data are scarce or expensive to gather, as is frequently the case in real-world circumstances, this technique is especially beneficial (See Algorithm 1).

In the realm of unsupervised or self-supervised representation learning, the Simple Contrastive Learning algorithm is a fundamental method. By contrasting pairs of samples, it is intended to capture significant aspects and relationships within the data. This approach has attracted a lot of interest across many fields, but especially in computer vision and natural language processing (see Algorithm 2).    
**Algorithm 1:** Self-Training for Semisupervised Image Classification**Input**: Labeled images, Unlabeled images**Output**: Trained Classifier**Initialization**:          Initialize a small set of labeled images;**Supervised Training**:          Train a classifier using the labeled images only;**Pseudolabeling**:          Use the trained classifier to predict labels for the unlabeled images;          Assign the most confident predictions as labels to the unlabeled images;**Augmented Training Set**:          Combine the labeled images with the newly labeled (pseudo-labeled) images. This creates a larger training set that contains both the initially labeled data and the newly labeled data;**Iterative Training**:          Combine the labeled images with the newly labeled (pseudolabeled) images;          Retrain the classifier using the augmented training set;**Validation and Testing**:          Monitor the model’s performance using a validation set or cross-validation;          Evaluate the final model on a separate testing set;

**Algorithm 2:** Simple Contrastive Learning algorithm [21]

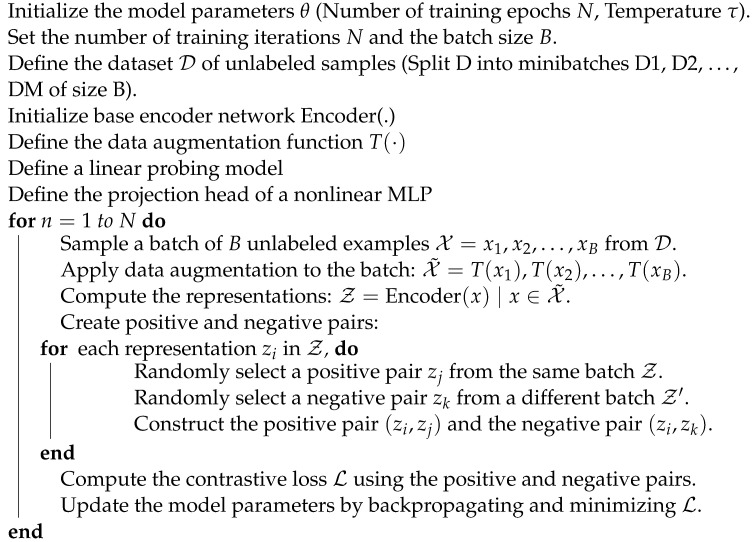



In this study, the contrastive learning framework is composed of the following:

★ Semisupervised

contrastive leaning:

There are 105,000 total images in the dataset used for contrastive pretraining-based semisupervised image classification, of which 100,000 are unlabeled and 5000 are tagged. Three divides are made from the dataset:

- Training Unlabeled: The encoder is trained in a contrastive context using this subset, which consists of 100,000 unlabeled images. Learning meaningful representations from the unlabeled data is the aim.
- Training labeled: A total of 5000 labeled photos make up the training labeled subset. It is employed to both fine-tune the pretrained encoder and train the baseline encoder under supervision. The tagged photographs offer further oversight to enhance the performance of the model.
- Testing Labeled: The models are assessed using this subset. It consists of labeled images and acts as a standard to evaluate the efficacy and accuracy of the models that have been trained using labeled data.

★ For self-supervised contrastive learning, the components of the framework are as follows:

- Data augmentation module: It generates two random augmentations for each input sample, each with a distinct view of the data and a subset of the original sample’s information.
- Encoder Network: Transforms the input sample ‘x’ into a representation vector ‘r’. The two augmented samples are supplied separately into the same encoder, producing a pair of representation vectors. ‘r’ is standardized to the hypersphere unit.
- Projection Network: Relates to the vector ‘z’. It can be implemented as a multilayer perceptron with a single hidden layer of 2048 and an output vector of 128, or as a single linear layer of 128. This network’s output is again normalized to the unit hypersphere, enabling distances in the projection space to be measured using the inner product method. These networks have been eliminated after the conclusion of contrastive training. As a result, our inference-time models have the exact same number of parameters as a cross-entropy model with the same encoder.

## 6. Results and Discussions

### 6.1. Contrastive Framework vs. Standard Data Learning Models

#### 6.1.1. Self-Supervised Learning

**Figure 8:**
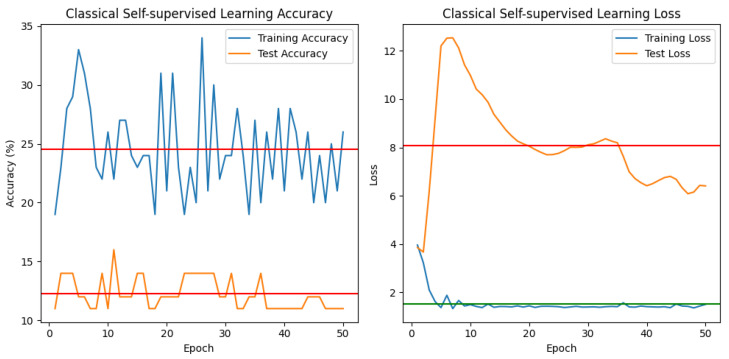
Traditional self-supervised model: The horizontal line for each curve reflects its average value.

★ Classical Self-Supervised training (See Figure 8).

To start with, we use a CustomDataset class that generates a synthetic dataset with randomly generated samples and labels. It provides methods for obtaining the length of the dataset and retrieving individual samples with their labels. The transform argument can be used to specify optional data transformations to be applied to the samples.

Based on the outcomes of training a self-supervised model with and without a contrastive approach for 50 epochs and assessing the related training and test losses, as well as accuracies, the following observations and conclusions can be made:

1. **Loss Fluctuations:** The training loss starts high at 3.8386 and drops throughout epochs, demonstrating the model is learning from the data. However, there are oscillations in the training loss over epochs, indicating that the training process may not be completely stable (see Figure 8).
2. **Accuracy Trends:** While accuracy percentages fluctuate, there is no consistent improvement across epochs. They swing within a specific range (e.g., 20–30%), with no distinct upward or descending trend, indicating potential problems with model convergence or optimization (see Figure 8).
3. **Overfitting Concerns:** Test loss tends to increase across epochs, whereas accuracy remains stable or declines slightly. This could imply overfitting, in which the model performs well on training data but fails to generalize to new test data, as indicated by falling or stagnant test accuracy (See Figure 8).
4. **Model Generalization:** Despite training for 50 epochs, test accuracy remained poor at 11–12%. This shows that the model is not properly capturing the underlying patterns in the data or that the model’s representation ability is limited (see Figure 8).
5. **Hyperparameter Tuning:** To improve performance, consider altering learning rates, regularization approaches, or model architecture. Experimenting with different configurations may aid enhance convergence and generalization (see Figure 8).
6. Further Analysis: Analyzing misclassified samples or inspecting model predictions can reveal specific issues. This could help identify areas for improvement or potential data biases.
7. **Evaluation Metrics:** Beyond accuracy, other metrics like precision, recall, or F1 score can provide a more thorough insight into a model’s performance, especially in imbalanced datasets.
8. **Data Quality:** The quality and representativeness of training data can considerably affect model performance. Having a broad and balanced dataset may aid enhance generalization.
9. **Continued Monitoring:** The model’s performance and conduct iterative training and evaluation cycles to resolve developing difficulties.
10. **Conclusion:** Although self-supervised training has potential, there are problems in getting satisfactory results. Addressing difficulties such as overfitting, model generalization, and hyperparameter tuning may improve the model’s performance in learning meaningful representations from data.

In conclusion, while the preliminary results show some progress, there is still much space for development, and a more extensive analysis, together with iterative testing and refining, is required to increase the model’s performance.

**Figure 9:**
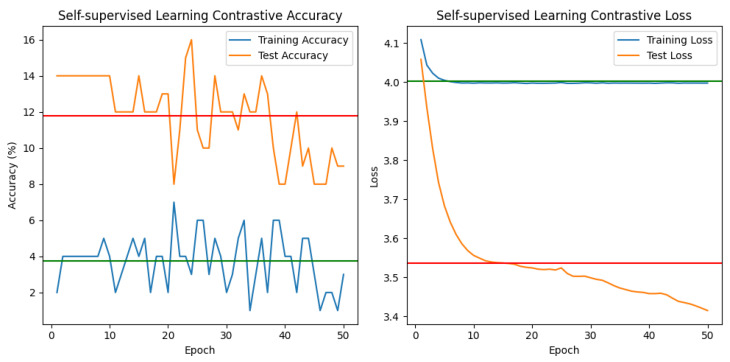
Self-supervised-contrastive model: Each curve’s horizontal line reflects its average value.

★ Contrastive Self-supervised learning (See Figure 9).

The supplied contrastive learning metrics demonstrate a model’s training and testing performance across 50 epochs. Here is a description of the patterns and trends seen:

1. **The “Contrastive Training Loss”** is the loss experienced during contrastive training, which tries to maximize agreement between positive pairs while minimizing agreement between negative pairs. The training loss lowers with each epoch, demonstrating that the model is learning to distinguish between similar and dissimilar cases. Throughout the training procedure, the contrastive training loss gradually falls from 4.1153 to 3.9973, demonstrating a shift toward acquiring more discriminative representations. Despite this drop, accuracy remains consistently low, ranging between 2.00% and 7.00%.
2. **The “Accuracy”** metric measures the percentage of properly anticipated cases during contrastive training. The training accuracy begins at 2.00% and gradually rises across epochs, reaching 6.00% in the final epoch. This indicates that the model is gradually strengthening its capacity to distinguish between positive and negative pairs.
3. **“Contrastive Test Loss”** refers to the loss calculated on a distinct test set. The test loss, like the training loss, diminishes with epochs, indicating that the model generalizes effectively to previously unseen situations
4. **The “Test Accuracy”** shows the accuracy achieved on the test set. The initial test accuracy is 0.0%, indicating low performance. However, it steadily improves and stabilizes at 10.00–15.00% after a few epochs. While this accuracy appears to be low, it is important to realize that contrastive learning accuracy is interpreted differently from typical supervised learning accuracy. In contrastive learning, the goal is to acquire meaningful representations rather than to complete a specific classification job. Despite some oscillations, the model’s training loss constantly reduces, showing that it is gradually learning to distinguish between positive and negative pairs.
5. **Convergence of training** and test losses implies effective learning without overfitting to training data.
6. **The model’s poor accuracy** scores indicate the need for additional fine-tuning or augmentation to increase discriminative skills. Overall, the model appears to be making progress in acquiring meaningful representations through contrastive training, as shown by decreasing loss values. However, the poor test accuracy suggests that there is still space for improvement in the model’s capacity to discern between positive and negative pairs. More experimentation and fine-tuning are required to attain better results.

In conclusion, while the contrastive learning model improves training loss over epochs, the low and changing test accuracy indicates difficulty in attaining successful generalization. Additional exploration and optimization may be required to solve these shortcomings and improve the model’s performance on previously unexplored data.

#### 6.1.2. Semisupervised Learning

In the context of semisupervised model learning, we are looking at two distinct models or techniques, with or without the use of the contrastive learning framework. The results obtained are particular to these two methodologies.

**Figure 10:**
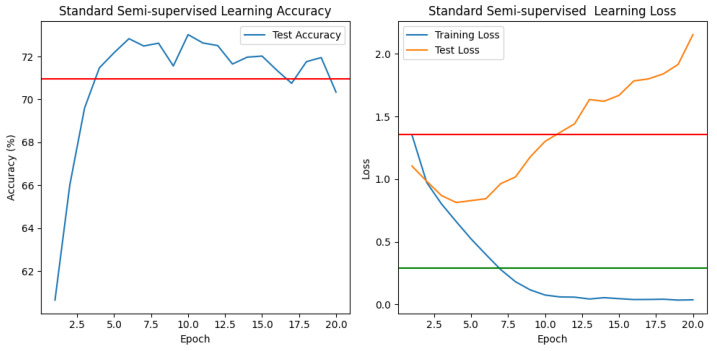
Classical semisupervised model: Each curve’s horizontal line denotes its average value.

★ Classical Semisupervised learning (See Figure 10).

In this scenario, we use the CIFAR-10 dataset, which includes 60,000 tiny color images. Each image has dimensions of 32 × 32 pixels. The dataset is divided into ten unique classes to ensure a balanced distribution of photos across the classes. Each class refers to a specific object or category.

1. **“The semisupervised train”** loss lowers from 1.3479 to 0.0381, suggesting effective learning from labeled and unlabeled data during training and parameter optimization to minimize the loss function.
2. **The test loss**, however, follows a distinct pattern. Initially, as the model learns more discriminative features from the training data, the test loss drops from 1.0898 to 0.8254 by epoch 5. This reduction indicates that the model is generalizing well to the test data, implying that the learned representations can be transferred to new samples. However, after epoch 5, we see a minor increase in test loss, which reaches 2.0129 by epoch 20. This increase in test loss indicates that the model’s performance on test data deteriorates as training advances. It implies that the model may be overfitting to the training data, collecting noise or specific patterns unrelated to the model’s generalization It suggests that the model is overfitting to the training data, capturing noise or specific patterns that are irrelevant to the model’s generalizability. The disparity between train and test loss indicates a potential overfitting problem. While the model gets decreased losses on the training data, indicating good fitting to the training set, its performance on the test data degrades, demonstrating an inability to generalize well to new samples.
3. **“The semisupervised test accuracy”** ranges from 60.82% to 73.00% in early epochs, then varies between 71 and 72%. This suggests that the model obtains decent accuracy on the test data but does not consistently improve it.

In the case of the semisupervised model, we see excessive overfitting, which is a phenomenon that occurs when the model becomes over-specialized in capturing the patterns and noise present in the training data, to the point where it becomes less effective at making accurate predictions on new data. This phenomenon can be seen visibly in the figure where the input error of the training data tends toward 0, while the error of the test data increases excessively.

**Figure 11:**
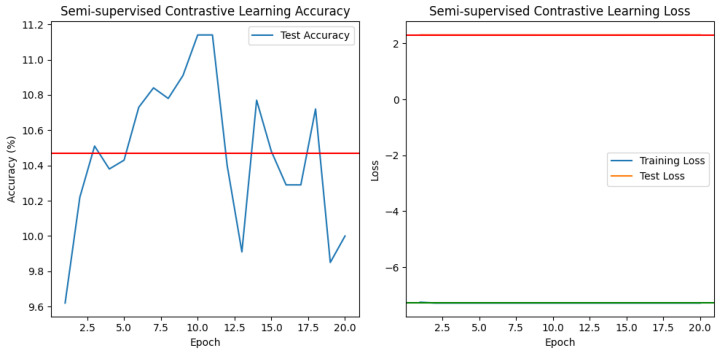
Semisupervised contrastive model: Horizontal line on each curve reflects the mean value.

★ Contrastive Semisupervised Learning (See Figure 11).

1. **“The contrastive train loss”** falls from −7.2641 to −7.2814 over epochs, showing that the model is learning to distinguish between similar and dissimilar samples in training data.
2. **Negative loss** refers to the loss of different or negative sample pairings. The goal is to reduce the resemblance or agreement among these negative pairs. As a result, the negative loss reflects the contribution of dissimilar pairs to the total loss function.
3. **“The contrastive test loss”** ranges from 2.3025 to 2.3027, indicating that the model’s performance on unseen data is not considerably increasing.
4. **“The contrastive test accuracy”** remains low throughout, ranging from 9.74% to 10.69%. This indicates that the model is struggling to accurately classify the test samples, as the accuracy is only slightly above random guessing (10%).

In the case of the traditional semisupervised model, we see excessive overfitting, which is a phenomenon that occurs when a machine learning model performs extremely well on training data but does not generalize well to new, unseen data. In other words, the model becomes overspecialized in capturing the patterns and noise present in the training data, to the point where it becomes less effective at making accurate predictions on new data. This phenomenon can be seen visibly in Figure 10, where the input error of the training data tends towards 0, while the error of the test data increases excessively.

In summary, the contrastive and standard semisupervised models have limited performance in terms of test accuracy and loss reduction. Further investigation of the data, model architecture, or training procedure may be required to uncover potential flaws and improve outcomes.

#### 6.1.3. Autosupervised Learning

**Figure 12:**
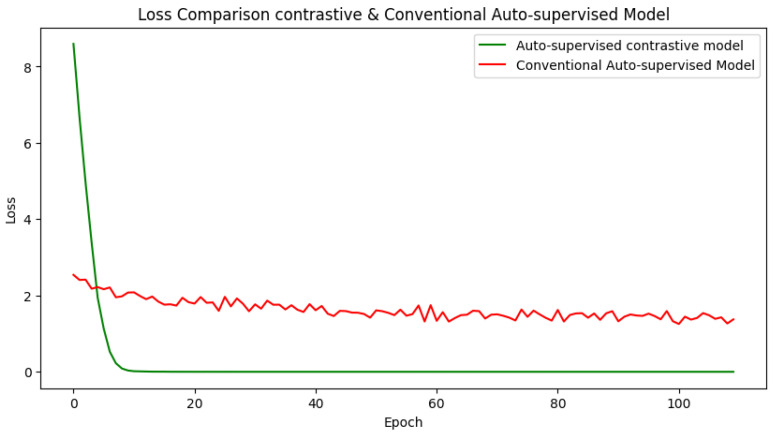
Loss comparison of contrastive and conventional autosupervised models.

★ Conventional Autosupervised and Contrastive Learning (See Figure 12).

The results of training the conventional autosupervised and contrastive learning models on the CIFAR-10 dataset for 100 iterations are shown in Figure 12. The figure depicts the loss values for both standard auto-supervised and contrastive learning frameworks, which span many epochs and learning stages.

For the autosupervised learning framework, the loss rapidly lowers over 10 epochs, demonstrating that the model is learning and improving. The initial loss is unusually significant at 2.5404, but it gradually falls to 1.3264, indicating that the model is adjusting to the training data and improving its prediction accuracy.

In contrast, the contrastive learning approach produces exceptionally low loss values. The loss begins at 8.5954 in the first epoch but swiftly drops to 0.0021 in the second epoch before reaching 0.0000 in the following epochs. These near-zero loss values demonstrate that the contrastive learning framework can effectively capture the similarities and contrasts between data, resulting in highly discriminative representations. The large difference in loss values between the two frameworks demonstrates the superiority of contrastive learning in this circumstance. The contrastive learning framework yields significantly lower loss values, implying that it is more capable of learning meaningful representations that capture the underlying structure of the data (see Figure 12).

### 6.2. An Experimental Comparison among the Three Contrastive Models

#### 6.2.1. Structural Similarity Index

The SSIM (Structural Similarity Index) [55,56] is a popular statistic for comparing the similarity of two photographs. It is intended to determine the perceived excellence of a distorted image in comparison to a reference image. The SSIM metric considers both structural data and the brightness similarity of the images. The SSIM index is calculated by comparing three variables: luminance (brightness), contrast, and structure. Mean, variance, and covariance values are used to evaluate each component. The individual element scores are then summed to generate the overall SSIM index, which runs from −1 to 1. The number 1 shows an exact correlation between the photos, whereas −1 indicates full dissimilarity [55,56].

(12) $$\mathrm{SSIM}\left(X,Y\right)=L{\left(x,y\right)}^{\alpha }\cdot C{\left(x,y\right)}^{\beta }\cdot S{\left(x,y\right)}^{\gamma }$$

where

(13) $$L\left(X,Y\right)=\frac{2\mu_{X}\mu_{Y}+C_{1}}{\mu_{X}^{2}\mu_{Y}^{2}+C_{1}}\;\left(Luminance\right)$$

(14) $$C\left(X,Y\right)=\frac{2\sigma_{X}\sigma_{Y}+C_{2}}{\sigma_{X}^{2}+\sigma_{Y}^{2}+C_{2}}\;\left(Contrast\right)$$

(15) $$S\left(X,Y\right)=\frac{\sigma_{XY}+C_{3}}{\sigma_{X}\sigma_{Y}+C_{3}}\;\left(Structure\right)$$

where

$$\left\{\begin{array}{c} C_{1}={\left(K_{1}\cdot L\right)}^{2}\\ C_{2}={\left(K_{2}\cdot L\right)}^{2}\;C1,\;C2,\;\mathrm{and}\;C3\;\mathrm{are}\;\mathrm{tiny}\;\mathrm{quantities}\;\mathrm{introduced}\;\mathrm{to}\;\mathrm{provide}\\ C_{3}=\frac{C_{2}}{2}\;\mathrm{numerical}\;\mathrm{stability}.\\ K_{1}=0.01,\;K_{2}=0.03,\;(L\;\mathrm{is}\;\mathrm{the}\;\mathrm{maximum}\;\mathrm{pixel}\;\mathrm{value}\;\mathrm{of}\;\mathrm{the}\;\mathrm{image},\;L\;=\;\\ \mu_{x},\mu_{y}\;\mathrm{represent}\;\mathrm{the}\;\mathrm{local}\;\mathrm{means}.\;\;255\;\mathrm{for}\;\mathrm{grayscale}\;\mathrm{photos}\;\mathrm{with}\;8\;\mathrm{bits}\;\mathrm{per}\;\mathrm{pixel}.)\\ \sigma_{x},\sigma_{y}\;\mathrm{represent}\;\mathrm{standard}\;\mathrm{deviations}.\\ \sigma_{xy}\;\mathrm{is}\;\mathrm{cross}-\mathrm{covariance}\;\mathrm{of}\;\mathrm{images}\;X\;\mathrm{and}\;Y.\\ \sigma_{x}^{2}\;\mathrm{is}\;\mathrm{the}\;\mathrm{variance}\;\mathrm{of}\;\mathrm{the}\;\mathrm{pixels}\;\mathrm{in}\;X\\ \sigma_{y}^{2}\;\mathrm{is}\;\mathrm{the}\;\mathrm{variance}\;\mathrm{of}\;\mathrm{the}\;\mathrm{pixels}\;\mathrm{in}\;Y. \end{array}\right.$$

If α=β=γ=1 (default for Exponents) and C3 = C2/2 (default selection of C3), the index becomes

(16) $$\mathrm{SSIM}\left(X,Y\right)=\frac{\left(2\mu_{x}\mu_{y}+c_{1}\right)\left(2\sigma_{xy}+c_{2}\right)}{\left(\mu_{x}^{2}+\mu_{y}^{2}+c_{1}\right)\left(\sigma_{x}^{2}+\sigma_{y}^{2}+c_{2}\right)}$$

#### 6.2.2. Floating Point Operations

**Supervised Contrastive Learning** (supervised contrastive learning considers different samples from the same class as positive examples, in addition to augmented versions).

**Self-Supervised Contrastive Learning** (makes use of the structure within the data to generate its own labels. It is trained on a pretext task + fine-tuning).

**Automated Supervised Contrastive Learning** (it learns representations from unlabeled data using a pretext task+supervised fine-tuning using a small amount of labeled data.

**Semisupervised Contrastive** (no self-supervised pretraining phase). The model learns to discriminate between labeled examples and also clusters similar unlabeled examples together based on their learned representations.

FLOPs, or Floating Point Operations, are a metric for the amount of computing in machine learning models. They track the amount of floating-point operations a model executes over inference or training. Understanding FLOPs can aid in model optimization for efficiency, particularly in contrastive learning, involving complicated structures and massive datasets. We used the Tensorflow profiler method to precisely calculate the number of floating-point operations (FLOPs) for each of our three trained models. This approach allowed us to avoid manually calculating FLOPs for individual layers, resulting in more precise observations. Table 2 provides a detailed breakdown of the FLOPs and attribute counts for all passes after training each model. These details shed light on the models’ computational complexity and provide information about the resources required for their particular processes. Using the Tensorflow profiler, we acquired consistent and meaningful measurements on FLOPs and parameter counts, allowing us to gain a better understanding of our trained models’ computational features.

The models were trained across 20 epochs, which is the number of times the complete training dataset was processed. The Adam optimizer, which is known for its ability to optimize stochastic gradients, was used to update the model’s parameters based on the computed gradients. This optimizer combines the advantages of adaptive learning rates with momentum to improve convergence speed and performance.

The calculations and computations were carried out using a hardware accelerator, particularly the T4 GPU. Because of its strong computing capability, the T4 GPU is a popular choice for machine learning workloads. It has a memory capacity of 12.7 GB, which allows for efficient data storage and retrieval during training. The T4 GPU’s enormous memory capacity allows it to handle huge datasets and complicated models, making training more efficient and effective.

### 6.3. Constructive Learning in Depth

We conducted several experiments to assess the effectiveness of the contrastive framework to traditional data learning models, with no optimization effort.

Now that we have established the contrastive model’s superiority over classical models, let us look at its characteristics (Data Augmentation, Objective Function, Encoders…) and how hyperparameters affect its behavior.

By investigating the contrastive model’s properties, we can see how it captures and represents information in training data. This will help us better understand its ability to develop discriminative representations and detect small distinctions between cases.

First, we take into account CIFAR-10 and MNIST, the benchmarks that are frequently utilized in contrastive learning research. In accordance with standard procedure, we whiten the datasets. To isolate the impact of the contrastive learning technique, we employ data augmentation, which is a machine learning technique for increasing the diversity and quantity of training data by applying various changes to existing datasets (see Section 3.2.1).

#### 6.3.1. Semisupervised Image Classification

Semisupervised image classification [35] is a method for categorizing huge numbers of unlabeled images when we have limited labeled data. Traditional supervised learning techniques need the compilation of a properly labeled dataset, which can be time-consuming and expensive. Semisupervised learning seeks to address this constraint by improving classification performance by using both labeled and unlabeled data. We start by thinking about contrastive pretraining using contrastive learning of visual representations for semisupervised image classification on the STL-10 dataset.

To successfully use deep learning in the real world, one typically has to collect a sizable dataset. Although labeling costs scale linearly with dataset size (labeling each sample requires a fixed amount of time), model performance only does so in a sublinear manner. As a result, labeling ever-increasing samples becomes less and less cost-effective, in contrast to the generally low cost of collecting unlabeled data due to its widespread availability.

By just requiring a partially labeled dataset and being label-efficient by using the unlabeled samples for learning as well, semisupervised learning offers a solution to this issue (see Algorithm 1). In this scenario, contrastive learning will be used to pretrain an encoder on the STL-10 semisupervised dataset with no labels at all, and only its labeled subset will be used for fine-tuning. During training, a large number of unlabeled images and a smaller number of labeled images will be loaded simultaneously.

#### 6.3.2. Characteristics of Contrastive Learning

The fundamental goal of contrastive learning is to develop self-supervised representations that are invariant to picture augmentations [5]. The fact that this objective has a trivial degenerate case in which the representations are constant and have no relation to the input images is a drawback.

Contrastive learning avoids this pitfall by altering the objective in the following way: it pushes various images away from one another in representation space (contrasting negatives) while simultaneously drawing representations of enhanced versions/views of the same image closer to one another (contracting positives).

Contrastive learning [6] is one such contrastive approach that essentially pinpoints the main elements required to maximize this goal and scales this straightforward strategy to attain high performance. The STL-10 dataset is an image recognition dataset used to research algorithms for deep learning, self-learning, and unsupervised feature learning. It draws inspiration from the CIFAR-10 dataset, albeit significant changes have been made. Each class offers fewer labeled training examples than in CIFAR-10, in particular, but a huge amount of unlabeled examples are provided to learn picture models prior to supervised training. The key difficulty is creating a usable prior using the unlabeled data, which come from a comparable but distinct distribution to the labeled data. Each image was obtained from a sample that was labeled on ImageNet. There are three divides in the dataset:

- Unlabeled Training: The encoder is trained using this dataset in a contrastive environment.
- Training Labeled: The baseline (supervised) encoder is trained using this dataset, and the pretrained encoder is also fine-tuned.
- Testing Labeled: The models are assessed using this dataset.

**Figure 13:**
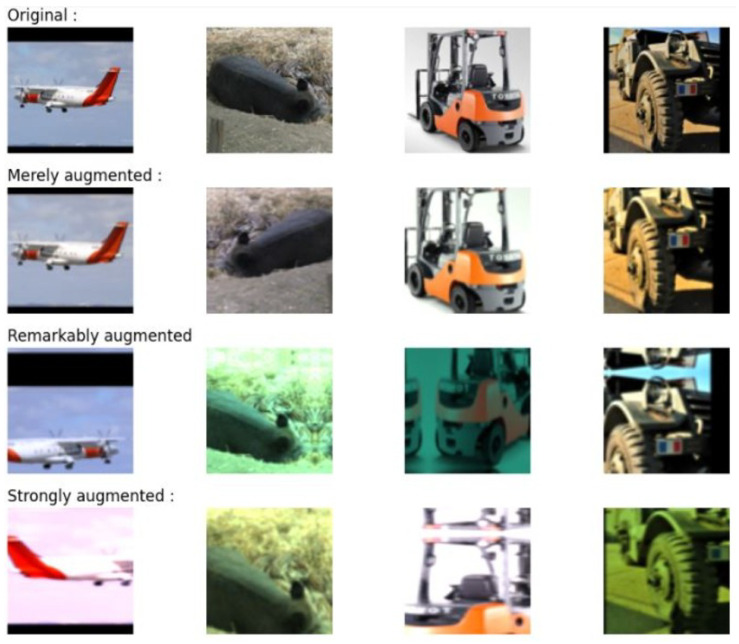
Images I confirme for moving the iamge contrasting augmentation reveals changes.

For contrastive learning, the following are the three most significant image augmentations (see Figure 13):

- **Color jitter** by distorting color histograms, hinders a simple color-histogram-based solution to the problem. Applying affine transformations in color space is a useful technique to carry that out.
- **Cropping an image** into smaller pieces forces the model to encode various portions of the same image similarly.
- **Contrastive zoom** is a method used to acquire a deeper understanding of the data. It entails comparing the differences or similarities between the data at various granularity or resolution levels.

To prevent overfitting on the few labeled samples, stronger augmentations are used for contrastive learning and weaker ones for supervised classification.

#### 6.3.3. Simulation Scenario: Contrastive Learning Pretraining for Semisupervised Image Classification

The initial test is conducted on the CIFAR-10 dataset [57] that has 6000 images per class and 60,000 32 × 32 color images in total. There are 10,000 test images and 50,000 training images. Each training batch contains 10,000 images, while the test batch contains 10,000 images. Each class’s 1000 randomly chosen photographs are included in the test batch. The remaining photographs are included in the training batches in a random order; however, some training batches may have a greater proportion of images from one class than another. Exactly 5000 photos from each class are included in each of the training batches combined.

- **Image augmentation:** The technique of developing new training examples from the ones that already exist is known as image augmentation [33,49]. It involves adding various transformations or perturbations to the original training images. We gently alter the original image to create a new sample. For instance, we might alter the original image to make it a little brighter, chop out a portion of it, or mirror the original image. The purpose of this technique is to boost the training data’s variability and generalizability, which can assist deep learning models to perform better and be more resilient [58]. This algorithm creates a series of nonspecific adjustments to the input photos as part of an image refinement process. Rescaling, horizontal flipping, random translation, random zooming, and custom color conversions are among the changes. The main goal of this sequence is to produce better training data that can increase a machine learning model’s generalizability and overall robustness during its training phase. In this example, we are creating code whose goal is to develop and build a pipeline for image augmentation. This technique is frequently used to enhance the generalization and resilience of machine learning models, particularly deep learning models, by artificially boosting the diversity of the training dataset.
- **Augmentation is used to display image contrasts**: By using the resulting model as the first stage in a deep learning pipeline, the incoming data can be enriched and diversified. The strength and inclusivity of the model will be increased as a result, which will ultimately increase its robustness and ability to generalize successfully (see Figure 13).
- **Encoder model design:** The next step is to define an encoder after applying the image augmentation technique to the dataset. The final model simulates an encoder network that takes an input image and flattens it into a lower-dimensional vector representation by applying numerous convolutional layers to it and then using this vector representation for various tasks like feature extraction or additional processing in a bigger neural network design [45].

The developed algorithm starts training a supervised model with a random initialization as its default setup. This technique serves as the foundation for the training process. The model’s parameters are randomly initialized to arbitrary values, introducing diversity from the start. These parameters are constantly modified throughout training based on input data and expected outputs, with the goal of improving the model’s performance on a specific job. This iterative approach focuses on lowering loss and improving the model’s prediction skills, which are frequently quantified using metrics such as accuracy. The baseline methodology enables a comparison of the model’s performance to various tactics or variants. The use of random initialization ensures that each model has a distinct beginning point, creating variation as the model advances. Once trained, this model can be used to make informed decisions regarding previously unseen data instances.

- **Supervised reference model:** The algorithm introduces a fundamental model in the context of supervised learning. This model serves as the baseline against which all subsequent comparisons and evaluations are made. It embodies a common and extensively used method in supervised learning studies. The baseline model’s principal function is to set a performance benchmark against which the efficacy of alternative models or approaches may be measured. The baseline model is trained using labeled data and specified features, and its performance may be measured using a variety of assessment criteria. This model is critical because it acts as a benchmark for comparing the relative benefits of more sophisticated models or advanced methodologies. This model is an important component that adds considerably to the evaluation and evolution of supervised learning algorithms, exposing the possible breakthroughs that may be achieved through invention and refining.

Using the Keras package, we construct a baseline model for picture categorization. A sequential model, which is a linear stack of layers, is specified as the model architecture. Our encoder architecture is a strided convolutional neural network that runs directly on the CIFAR-10 dataset, which contains 60,000 32 × 32 color images. Four convolutional layers with strides [2, 2, 2, 2], kernel sizes [3, 3, 3, 3], and 128 hidden units are used. The rectified linear unit (ReLU) is the activation function utilized in these convolutional layers.

A layer follows the convolutional layers. The layer Flatten() is added. In preparation for the following fully connected layers, it flattens the output of the convolutional layers into a 1D vector.

**Figure 14:**
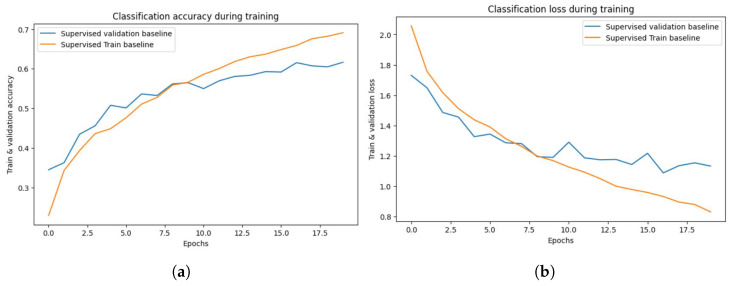
Supervised baseline model accuracy/loss: (**a**) Shows convergence of model accuracy for the supervised baseline model. (**b**) Demonstrates model loss for the supervised baseline model.

Finally, a layer called Dense() is introduced to represent a fully connected layer. The number of neurons in this layer is determined by the width parameter. ReLU is the activation function employed in this layer. We train on a subset of the CIFAR-10 dataset. The Adam optimizer is used. During training, we will use a loss function to calculate the difference between the model’s expected and actual output. This loss function includes the Sparse Categorical Accuracy metric, which quantifies the model’s prediction accuracy in comparison to the real labels. The T4 GPU is designed to provide high-performance computing capabilities, which will be used in the training procedure, with a minibatch size of 500 for unlabeled photos and 25 for tagged images. Based on these minibatches, the accuracy and loss values will be computed, as shown in Figure 14a,b, respectively.

The findings (see Figure 14) demonstrate the evolution of the model’s performance over each training epoch. The training loss and accuracy show how well the model fits the training data, whereas the validation loss and accuracy show how well the model generalizes to previously unseen validation data (see Table 3).

#### 6.3.4. Contrastive Pretraining Model

The Algorithm 2 employs a self-supervised contrastive loss that seeks to learn meaningful representations by encouraging similar representations for enhanced views of the same image and dissimilar representations for augmented views of different images. A contrastive accuracy metric is used to monitor the similarity estimation’s correctness [36]. The algorithm assumes that the inputs consist of feature vectors derived from two augmented perspectives of an initial image. These perspectives are generated by applying data augmentation methods to the original image. The contrastive loss is then determined using the similarity scores. The representations of two augmented perspectives of the same image in this self-supervised learning setup ought to have greater similarity scores than the representations of other images. In order to enforce this, the loss is created in a way that separates representations of negative pairings (from different images) while bringing those of positive pairs (from the same image) closer together. The algorithm denotes the definition of a linear probing model using a single dense layer of 10 units.

The algorithm then specifies a nonlinear projection head using an MLP (multilayer perceptron) network of dense two-layer neurons. The first dense layer employs a ReLU activation function to introduce nonlinearity, but the second dense layer is linear by default.

Table 4 displays experimental results for a self-supervised model applied to the CIFAR-10 dataset, with metrics such as training accuracy at 78.43% and validation accuracy at 57.48%. These metrics represent the model’s performance, which is acceptable during training and evaluation in the case of the self-supervised contrastive learning model.

#### 6.3.5. Supervised Fine-Tuning of Pretrained Encoder

**Figure 15:**
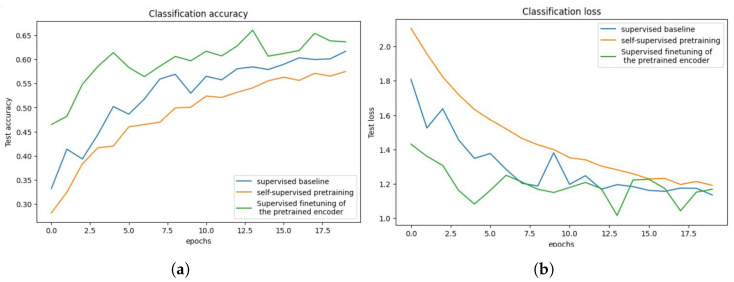
Baseline, self-supervised, and fine-tuning model accuracy/loss: (**a**) Demonstrates higher validation accuracy for baseline, self-supervised, and fine-tuning models. (**b**) Shows lower validation loss for baseline, self-supervised, and fine-tuning models.

By examining the training curves of the contrast pretraining model, we may see significant improvements in validation accuracy and loss. Figure 15 illustrates that the contrast pretrained model outperforms the baseline model in terms of validation accuracy (see Figure 15a), and shows how the contrast pretrained model also has lower validation loss (see Figure 15b). These findings imply that the pretrained network can perform better overall even with fewer labeled data available.

The contrast pretraining method entails training a model using self-supervised learning methods [36], particularly contrastive learning. By contrasting positive and negative pairings of examples, this method enables the model to learn meaningful representations. In the context of this study, it is presumable that the inputs to the contrast pretrained model are the output feature vectors of two enhanced views of an input picture. These views are created by using data augmentation techniques on the source image. The model is trained to maximize similarity between positive pairings (examples that should be similar) while limiting similarity between negative pairs (examples that should be dissimilar) during the contrast pretraining phase. This enables the model to recognize and comprehend the fundamental patterns and structures in the data, leading to more inclusive representations.

When analyzing the training curves, the advantages of contrast pretraining become clear. The contrast pretrained model’s improved validation accuracy shows that it has acquired more robust and discriminative features. It performs better than the baseline model, which most likely went through conventional supervised training without the advantage of contrastive pretraining.

Additionally, the contrast pretrained model’s lower validation loss suggests that it is better suited to handle situations with few labeled data. This displays the pretrained network’s generalization ability as it successfully applies the learned representations to produce precise predictions even in the absence of sufficient labeled data.

The results imply that comparison pretraining is an effective strategy for improving the functionality of machine learning models. The model may develop rich representations that capture significant features and patterns in the data by making use of self-supervised learning and contrastive approaches. The model can now attain greater accuracy and less loss, which shows enhanced generalization and resilience.

In conclusion, using a contrast pretrained model increases validation accuracy and decreases validation loss, showing that the model generalizes effectively even with few labeled samples. This demonstrates how contrast pretraining can improve the functionality and generalizability of machine learning models.

### 6.4. Supervised Contrastive Learning

Supervised contrastive learning (SCL) is a technique used in machine learning for representation learning that is a version of contrastive learning [59,60]. The similarity or dissimilarity of samples is defined by the class labels or annotations associated with them in supervised contrastive learning.

The core concept of supervised contrastive learning is to use class labels to guide the learning process and improve the discriminative strength of the acquired representations. The goal of supervised contrastive learning is to bring samples from the same class closer together in the learned representation space while pushing samples from different classes further away by using class labels (See Figure 4).

In supervised contrastive learning, the training method often entails constructing positive and negative pairs of data. Positive pairs are examples from the same class, whereas negative pairs are samples from separate classes. The model is then trained to maximize positive pair agreement while minimizing negative pair agreement.

**Figure 16:**
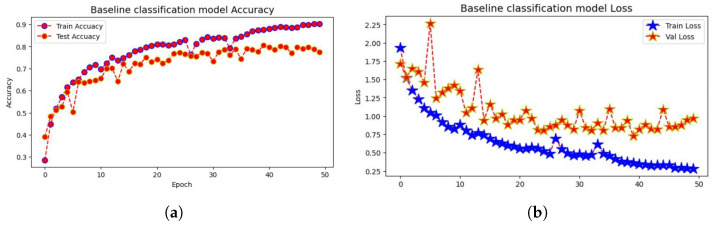
Supervised baseline model accuracy/loss: (**a**) Demonstrates model accuracy convergence over training and validation data. (**b**) Demonstrates model loss for baseline classification model.

As shown in Figure 16a,b, the information supplied describes the evolution of a neural network model’s accuracy and loss during the training period, respectively. The model is trained across 50 epochs, each with numerous batches.

The model obtains a trainong accuracy of 91.31% and test accuracy of 81.23% after 50 epochs of training (see Table 5).

After fifty iterations, the considerable difference in training and validation for accuracies and losses, as seen in Figure 16, is strong evidence of overfitting, which increases with the number of iterations. When a model learns to perform well on training data but fails to generalize to new, unseen data, this is referred to as overfitting. The divergence in training and validation loss in later epochs indicates that the model is beginning to memorize the training data rather than learning significant patterns [61].

Overall, the model’s accuracy is increasing, demonstrating that the model’s performance improves as training advances. It is important to note, however, that accuracy does not necessarily rise monotonically and may display significant variations during the training process.

#### 6.4.1. Pretrain the Encoder

The goal of pretraining the encoder is to equip the model with the ability to learn valuable features or representations of the input data without the need for a particular task or labeled examples. The encoder can learn to recognize patterns, semantics, and other linguistic characteristics of the language by being trained on a lot of text data.

As with the previous model, the pretrained encoder model’s loss gradually decreases over the course of the 50 epochs, indicating that the model is learning and improving its performance on the training data.

#### 6.4.2. Train the Classifier with the Frozen Encoder

A pretrained model, such as an image feature extractor, is referred to as a frozen encoder when the model’s weights are fixed and unchanging during the training of the following classifier. When we want to use the learnt features of a strong pretrained model while only improving the classifier component, this approach is advantageous.

The broad breakdown of the processes we use is as follows:

- **Create a classifier** model that includes a fresh classification layer after the frozen encoder. The phrase "frozen encoder" refers to an encoder whose parameters and weights do not change throughout training.
- **Select a loss function** like the cross-entropy loss that is appropriate for the classification task. Additionally, decide on an optimizer (like Adam) to modify the classifier’s weights throughout training. Process the training data in batches during training and then run the input data through the frozen encoder to obtain encoded representations. Put these encoded representations into the classification layer after that, then calculate the loss. To be more precise, we hold the encoder in a frozen state while propagating the gradients through the classifier section, updating its parameters and weights.

To monitor the model’s development, periodically assess its performance on the validation dataset.

Use the test dataset to evaluate the final model’s performance after the training phase is complete to obtain a fair assessment of its performance.

This method makes it easier for the model to adjust and improve the encoder’s representations to match the subtleties of our particular classification task.

**Figure 17:**
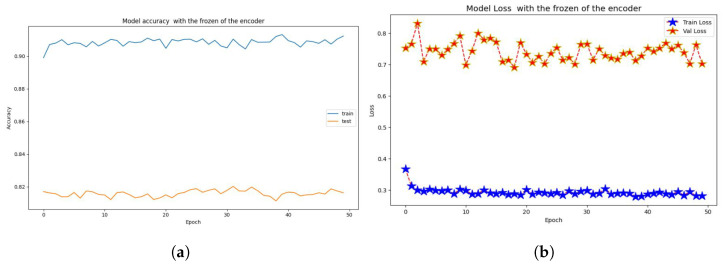
Frozen encoder model accuracy/loss. (**a**) Demonstrates model accuracy convergence with a frozen encoder. (**b**) Demonstrates model loss convergence with a frozen encoder.

Overall, it seems like the training process is working. Over the course of the training epochs, the loss values go down (See Figure 17b) and the sparse categorical accuracy values go up (See Figure 17a). Additionally, it appears that over the training period, the model’s performance on the validation dataset has improved. The model is able to make reasonably accurate predictions on fresh, untested data, as seen by the test accuracy of 82.02% (see Table 6). However, it is crucial to keep an eye on the metrics for validation and take into account additional strategies like regularization if the validation accuracy plateaus or declines.

According to Figure 17, the training data demonstrates the performance of a neural network model over a period of 50 epochs. The architecture of the model was trained on a dataset, and its performance was assessed on both the training and validation sets. Here’s a rundown of the main points: The validation loss began at 0.80 and fluctuated throughout training but stayed within a defined range. Similarly, the sparse categorical accuracy on the validation set began at 0.81 and fluctuated around this value during training, demonstrating slight gains. With oscillations in the training process, the model appears to have reached a certain level of accuracy on both the validation and test sets.

Regardless of fluctuations during the training phase, the model maintained a reasonable level of accuracy on both the validation and test sets until fine-tuning began. Surprisingly, only the early epochs saw an increase in training accuracy. Many explanations exist for this behavior. For starters, the pretrained model could appropriately categorize a sample of the data, which aided its early performance on the validation and test sets. Second, following the first learning phase, the model may begin to overfit to the training data by retaining specific examples as opposed to learning generalizable patterns [61]. This can cause training accuracy to stagnate or even drop, while the model’s efficacy on unseen data (validation or test set) could keep improving. Third, due to the spread of data, the initial boost in accuracy could be attributed to the unique distribution of the training data, in which the model quickly learns to exploit patterns in the training set but fails to generalize to previously unseen samples. Fourth, the model may not be sufficiently complex to capture the dataset’s full complexity. While it may perform well on simple samples at first, it has difficulty generalizing to increasingly challenging cases. Although the model gained some accuracy early on, additional analysis is required to evaluate its overall performance, including metrics such as precision, recall, and F1 score. Further advancements may necessitate investigating changes to the model architecture, data preprocessing, or training methodologies.

#### 6.4.3. Fine-Tune

**Figure 18:**
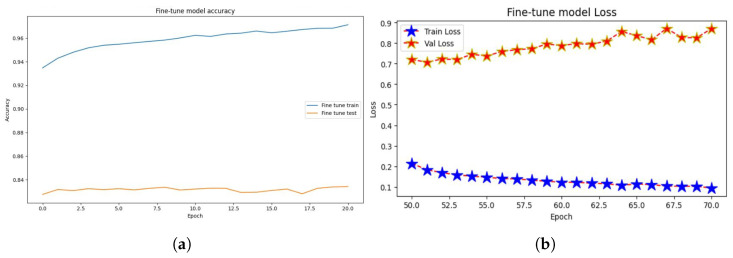
Fine-tuned model accuracy/loss: (**a**) Demonstrates model accuracy convergence. (**b**) Demonstrates model loss convergence.

The performance of a neural network model is shown over the course of 20 epochs in these fine training results, along with related validation and test metrics (See Figure 18). The main observations are broken down as follows:

- **Accuracy:** The training set’s sparse_categorical_accuracy increases from 0.93 to 0.97 (See Figure 18a), showing that the model is capable of correctly classifying samples. A similar improvement in the validation accuracy (val_sparse_categorical_accuracy) from 0.82 to 0.84 (see Figure 18a) suggests that the model generalizes effectively to new data.

- **Loss Reduction:** As epochs pass, the training and validation losses go smaller, demonstrating that the model is improving with each iteration at fitting the data. It can be inferred from this that the model is successfully reducing its prediction errors.
- **Generalization:** There may be some overfitting, but the difference between training and validation accuracy/loss is not that different, and the model performs marginally better on the training set. If necessary, regularization methods like dropout or weight decay could be taken into consideration to reduce overfitting.
- **Test Results**: The accuracy of the final test was 83.42%, which is in line with the accuracy of the validation (See Figure 18b). This shows that the model’s performance on unobserved data (test set) and performance on the validation set are comparable, which implies that the model is well-generalized.
- **Consistency:** Across epochs, increases in accuracy and loss are typically consistent and devoid of large oscillations. This implies that the training process is steady and advancing forward.
- **Convergence Speed:** It appears that the model’s convergence speed is appropriate. As training progresses, the accuracy and loss improvements start to plateau, indicating that the model has reached a point where further improvements may call for further training epochs or changes to the model’s design.

The bottom line is that these outcomes show a well-trained model that is performing fairly well on both the validation and test datasets. Depending on the application, more analysis or tweaking may be performed to improve the model’s performance or comprehend its behavior.

Overall, the data indicate that the model’s performance plateaued and did not increase appreciably in subsequent epochs. Further investigation could include looking into the model’s performance on previously unseen data, potential overfitting prevention measures, and experimenting with hyperparameter adjustments to improve model training. The training method we described entails fine-tuning a model, which results in a training accuracy of 96.95% and a validation accuracy of 83.17% (see Table 7). Furthermore, the losses are modest, showing that the learning is successful. This indicates that the model has learned to match the training data effectively and is also performing well on unseen validation data. The outcomes are excellent, demonstrating the efficacy of the fine-tuning process. Maintaining such a high train accuracy while simultaneously attaining a significant validation accuracy shows that the model has not only mastered the training data but has also been able to generalize to new samples. The reduced losses further verify the fine-tuning process’s usefulness in increasing the model’s performance. This result reflects a meticulously controlled fine-tuning method, which most likely included rigorous hyperparameter tweaking and regularization strategies to avoid overfitting. Overall, the combination of high train accuracy, acceptable validation accuracy, and minimal losses shows a successful fine-tuning effort that has resulted in the model’s capabilities being enhanced.

These findings demonstrate the efficacy of supervised contrastive learning in improving the model’s discriminatory abilities. During fine-tuning, the model learns to better differentiate between distinct classes, resulting in increased accuracy on both the training and validation sets. The large rise in training accuracy indicates that the model successfully captures the differentiating properties of each class, allowing for reliable categorization. The observed increase in validation accuracy implies that the model’s learned representations adapt effectively to previously unknown data, demonstrating its capacity to generalize beyond the training dataset.

### 6.5. Contrastive Pretraining Using Linear Classification

In this example, we use the contrastive learning framework to perform contrastive pretraining on the MNIST dataset. After that, we refine a linear classifier using the features learned by the pretrained encoder. We show the training and validation methods for both phases and encompass features such as model storage, retrieval, learning rate modifications, and the potential inclusion of Earlystopping [61].

In this scenario, we employ a model with contrastive pretraining, which is an unsupervised learning strategy in which a model is trained to distinguish between similar and dissimilar pairs of data points. It entails teaching a model to generate meaningful data representations by predicting the similarity or dissimilarity of pairs of samples.

**Figure 19:**
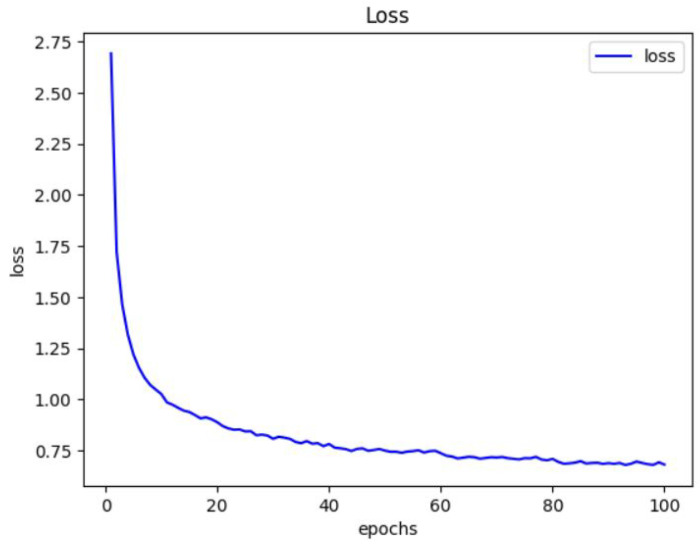
Demonstrates decreasing learning rate helps the model converge to an optimal solution.

We can observe in the graph that the loss lowers with each epoch after training this model. This shows that the model is learning to differentiate between data points and improving its feature representations. By epoch 100, the loss had dropped dramatically to 0.69, indicating that the model had learned to encode the data in such a way that it could efficiently distinguish between positive and negative pairings. The loss is decreasing steadily, showing that the model is learning and refining its feature representations. The decreasing learning rate aids the model in reaching a satisfactory solution (see Figure 19).

**Figure 20:**
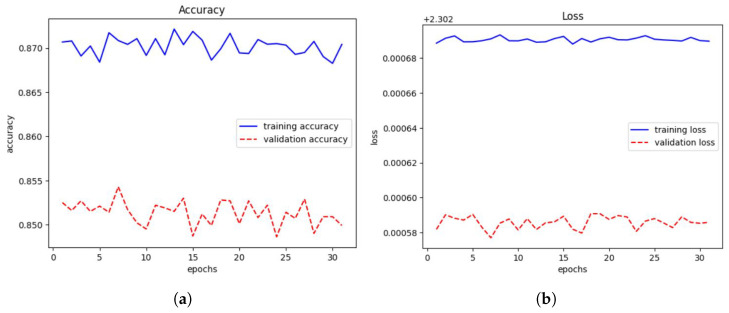
Convergence of linear model accuracy/loss: (**a**) Demonstrates the convergence of linear model to an optimal solution for accuracy. (**b**) Demonstrates the convergence of linear model to an optimal solution for loss.

The findings are depicted in Figure 20 as the progression of a model’s training and validation performance throughout multiple epochs. The major parameters being monitored include both accuracy (see Figure 20a) and loss (See Figure 20b). The accuracy scores for both training and validation are remarkably stable, staying around 86.78–87.22% for training and 84.88–85.43% for validation. Early stopping, which is often centered on the monitoring of validation loss, entails ceasing training when either improvements or regressions begin. In this case, the early stopping counter is increasing, indicating that the model’s performance on the validation set is stagnant or declining. The consistency of loss and accuracy measurements could be attributed to a variety of reasons. The model may be struggling with convergence issues or may have reached a shallow solution. Alternately, it is possible that changes to the model’s architecture or hyperparameters are required.

### 6.6. Specific Architectural Design Using Multilayer Perceptron (MLP)

Experimentation and model selection based on task-specific performance are frequently required to discover which type of model is optimal for a certain contrastive learning challenge. In addition, fine-tuning may be required to attain the best results. In this scenario, we change a few parameters to increase the performance of our model. The first is the type of architectural projection head. A linear transformation that translates coded representations onto a lower dimensional space is typically used as the linear head. The values obtained after training the model with this parameter are shown in the Table 8.

We can replace the linear head in our contrastive learning architecture with a multilayer perceptron by adjusting the parameter of the MLP (multilayer perceptron) head. This parameter allows nonlinear transformations to be applied to encoded representations, which has the ability to capture subtle patterns and relationships. Figure 9 illustrates the results obtained after training the model and shows how we can improve the performance of our contrast learning architecture.

When the accuracies of the two models are compared, it appears that the model MLP performs better (79.14% in favor of the model MLP versus 76.50% for the model linear) (see Table 9). This could be explained by the fact that linear models are simple and have a linear decision boundary. They can capture linear relationships in data effectively, but they may struggle with more complex, nonlinear correlations. Linear models can be employed as embedding functions in contrastive learning to project data into a lower-dimensional space where the contrastive loss is applied. Linear model embeddings are often more interpretable because they are closely related to the input features.

MLPs, on the other hand, are neural networks with numerous layers that include nonlinear activation functions. MLPs are capable of learning complex, nonlinear data correlations (see Table 9). They can simulate more sophisticated representations by capturing detailed patterns and hierarchies. MLPs can be employed as the embedding function in contrastive learning to transform input data into a learned representation. MLPs’ nonlinear activation functions allow them to capture complex correlations between input features.

Other hyperparameters may increase performance. Indeed, after changing the temperature parameter value and training numerous times, the model achieves a high degree of accuracy and maintains constant loss values throughout a wide range of temperature settings (the best score for accuracy is 88.77% for the training dataset and 88.70% for the validation dataset) (see Table 10). However, the precise temperature chosen can be determined by the desired trade-off between training and validation accuracy in a given application (see Table 10).

The use of contrastive learning (MLP model) on the MNIST dataset demonstrates the efficiency of contrastive pretraining in creating meaningful representations of data. Throughout the training process, the model’s loss is continually reduced, demonstrating superior feature discrimination. This highlights the value of contrastive pretraining in improving representation learning. The model’s performance on the MNIST dataset demonstrates the efficacy of contrastive pretraining in improving representation learning.

In the case of the last experiment (see Table 10), the optimum value for the highest accuracy (88.77%) is τ = 0.075.

Here is a more detailed explanation of when to utilize low and high temperatures. Low Temperature: A temperature setting to low values will make the probability distribution more pronounced. In other words, the expected odds for the positive and negative samples diverge significantly. The model gains increased assurance in its ability to discriminate between positive and negative samples. In situations where it is relatively simpler to distinguish between the positive and negative samples or when there is less intrinsic noise in the data, using a lower temperature is frequently preferable. It can result in more discriminative representations by motivating the model to concentrate on drawing more accurate distinctions. When it comes to differentiating between positive and negative samples, the model gains confidence. In addition to potentially producing more discriminating representations, it motivates the model to concentrate on drawing more exact differences.

High Temperature: The probability distribution is smoothed when the temperature is set to a higher value. This indicates that there is an increasing similarity between the expected probabilities for the positive and negative samples. In other words, the model loses confidence in its ability to discern between positive and negative samples. When it is difficult to distinguish between the positive and negative samples or when there may be inherent noise or uncertainty in the data, using a higher temperature can be helpful. It facilitates a more exploratory learning style and keeps the model from being too confident.

It is important to recognize that a model’s accuracy might vary greatly based on a variety of factors, including the features of the dataset, the nature of the task, the model architecture chosen, and other relevant considerations. To provide more information, here are a few prominent examples that serve as illustrations:

In a study of image classification using contrastive learning [20] (see Section 2), a ResNet-50 model trained with contrastive pair learning attained a top-1 accuracy of 81.68% on the on CIFAR-100 dataset.

In the study cited in [7] (see Section 2), the methodology entails using self-supervised contrastive learning techniques and adjusting them for supervised classification by integrating label information and computing the contrastive loss using a projection network. The top-1 accuracy attained in supervised contrastive learning using a cross-entropy loss on the ImageNet dataset with the ResNet-200 architecture is 81.4%.

In the study in [32] (see Section 2), the research discusses Image Enhanced Rotation Prediction, a unique pretext task for self-supervised learning (SSL). It also contrasts Rotation and supervised pretrained models as baselines. The pretrained dataset was ImageNet, and the feature extractor architectures used were ResNet-50, R-CNN, and Mask R-CNN. In the best-case situation, the accuracy was more than 80.3%.

The article in [8] (See Section 2) employs the Barlow Twins technique, which seeks to learn representations by maximizing cross-correlation between enhanced views of the same image while minimizing cross-correlation across augmented views of various images. By removing batch-normalization methods from the two hidden layers of the projector network MLP, the highest top-1 accuracy is around 71.2%.

## 7. Limitations

Despite its accomplishments, our work has some limitations, including the following:

- The effectiveness of our strategy is dependent on the use of contrastive learning, which demands significant data augmentation and a large number of negative samples.
- While fine-tuning is a typical strategy in contrastive learning that frequently results in increased performance on subsequent tasks, its theoretical basis and full degree of advantages remain uncertain.
- As demonstrated in the last scenario, the sensitivity to hyperparameters Contrastive learning algorithms are sensitive to hyperparameters, which can be difficult to accurately adjust. Inappropriate hyperparameters can cause the algorithm to perform poorly or even fail entirely.

## 8. Conclusions

As demonstrated, contrastive learning can improve both the accuracy and robustness of classifiers, which is an important improvement for many applications.

To summarize, experimentation and choice of models based on task-specific performance are critical in contrastive learning. Based on the experimental results, the neural network model, which was fine-tuned via supervised contrastive learning, shows promising performance. The training accuracy goes from 93% to 97%, suggesting that the model can accurately categorize samples. The validation accuracy rises from 82% to 84%, showing effective generalization. The training and validation losses are decreasing, showing that the model is improving and lowering prediction errors. The contrastive learning framework, when applied to the MNIST dataset, demonstrates the effectiveness of contrastive pretraining in learning meaningful data representations. The model’s loss decreases with each epoch, indicating improved feature differentiation. By Epoch 100, the model has a loss of 0.69, indicating that data pairs are efficiently encoded and discriminated. The training and validation accuracies are similar, at around 86.78–87.22% and 84.88–85.43%, respectively. Significant performance gains can be made by fine-tuning aspects like the type of architectural projection head. In this scenario, substituting the linear head with a multilayer perceptron (MLP) improved accuracy (79.14% for MLP vs. 76.50% for linear). MLPs, with their nonlinear activation functions, may detect complicated correlations and patterns. Furthermore, changing hyperparameters such as the temperature parameter can improve accuracy, with the top score being 88.77% for the training dataset and 88.70% for the validation dataset. The temperature is chosen based on the desired balance of training and validation accuracy.

The results presented in this research have the potential to improve accuracy in a variety of computer vision applications, particularly when additional data coding is costly or difficult. This can be very beneficial in areas such as reorganization (clustering): it can be used to group together similar data based on their embeddings, which can be useful in data analysis and the discovery of hidden structures. In the field of information search, contrastive learning can be used to improve similarity-based search results. The models are compelled to encode the data in such a way that similar elements can be grouped together and easily found during a search. In the field of content recommendation, contrastive learning can be used to learn different representations of articles, products, or users. The models learn to represent similar elements in the space of characteristics in a more accurate manner, which can be used to recommend similar articles or products to a given user. In medical applications, where the precise annotation of medical data necessitates the engagement of clinical professionals, and where semisupervised learning approaches could have a significant impact on the lives of patients. For object detection, contrast learning can help improve object detection precision by eliciting more robust characteristics.

Overall, contrastive pair learning appears as an essential technique in the field of representational learning and image classification, providing efficient learning, superior feature discrimination, and competitive performance across a wide range of datasets and tasks.

Future studies could explore pipeline adaptation to include transition regions across the two converted images, similar to the binocular region of the human field of vision. In addition, we plan to explore the new potential of contrastive learning in image models, as well as other reduction approaches for dataset augmentation.

Fine-tuning increases contrastive learning, although its theoretical roots and effects are uncertain. Further research is required to further understand its effectiveness and potential improvements.

## Figures and Tables

**Table 2:** Count of FLOPs and parameters for every model.

Model	FLOPs (M)	Trainable Params (K)
Automated Supervised Contrastive	157.793	20.490
Self-Supervised Contrastive learning	41.648	157.578
Semisupervised Contrastive	626.805	5.130

**Table 3:** Supervised baseline model: max accuracy/min loss.

Dataset	Train Accuracy (%)	Validation Accuracy (%)	Train Loss	Validation Loss
CIFAR-10	71.14%	61.68%	0.82	1.14

**Table 4:** Self-Supevised Model: Max Accuracy/Min Loss.

Dataset	Cons. Train Accuracy (%)	Cons. Train Loss	Probe Accuracy (%)	Probe Loss	Validation Accuracy	Validation Loss
CIFAR-10	78.43%	1.35	57.72%	1.16	57.48%	1.19

**Table 5:** Baseline: Accuracy/Loss.

Dataset	Train Accuracy (%)	Test Accuracy (%)	Train Loss	Test Loss (%)
CIFAR-10	91.31%	81.23%	0.25	0.76

**Table 6:** Model accuracy and loss with the frozen of the encoder.

Dataset	Train Accuracy (%)	Test Accuracy (%)	Train Loss	Test Loss (%)
CIFAR-10	91.32%	82.02%	0.28	0.69

**Table 7:** Fine-tune model.

Dataset	Train Accuracy (%)	Test Accuracy (%)	Train Loss	Test Loss (%)
CIFAR-10	96.95%	83.17%	0.09	0.71

**Table 8:** Fine-tuned model (Head_type = linear).

Dataset	Train Accuracy (%)	Test Accuracy (%)	Train Loss	Test Loss (%)
MNIST	76.50%	74.81%	2.30	2.30

**Table 9:** Fine-tuned model (Head_type = MLP).

Dataset	Train Accuracy (%)	Test Accuracy (%)	Train Loss	Test Loss (%)
MNIST	79.14%	79.09%	2.30	2.30

**Table 10:** Fine-tuned model accuracy and loss with different values of temperature (Head_type = MLP: The bold component highlights the fluctuation in accuracies, which peak at a particular temperature).

Temp.	Dataset	Train Accuracy (%)	Validation Accuracy (%)	Train Loss	Validation Loss (%)
0.007	MNIST	78.05%	79.22%	2.30	2.30
0.01	MNIST	81.25%	80.88%	2.30	2.30
0.04	MNIST	81.89%	82.03%	2.30	2.30
0.06	MNIST	82.32%	82.83%	2.30	2.30
**0.07**	MNIST	**87.22%**	**85.43%**	2.30	2.30
**0.075**	MNIST	**88.77%**	**88.70%**	2.30	2.30
**0.08**	MNIST	**85.65%**	**84.96%**	2.30	2.30
0.10	MNIST	79.88%	80.29%	2.30	2.30
0.15	MNIST	79.00%	78.59%	2.30	2.30
0.2	MNIST	80.22%	81.30%	2.30	2.30
0.3	MNIST	81.91%	82.16%	2.30	2.30

## Data Availability

Popular image classification datasets are benchmarked. MNIST: Official website: http://yann.lecun.com/exdb/mnist/; CIFAR-10: Official website: https://www.cs.toronto.edu/~kriz/cifar.html; STL-10: Official website: https://cs.stanford.edu/~acoates/stl10/, (accessed on 3 July 2024).
